# A Broad Phenotypic Screen Identifies Novel Phenotypes Driven by a Single Mutant Allele in Huntington’s Disease CAG Knock-In Mice

**DOI:** 10.1371/journal.pone.0080923

**Published:** 2013-11-22

**Authors:** Sabine M. Hölter, Mary Stromberg, Marina Kovalenko, Lillian Garrett, Lisa Glasl, Edith Lopez, Jolene Guide, Alexander Götz, Wolfgang Hans, Lore Becker, Birgit Rathkolb, Jan Rozman, Anja Schrewed, Martin Klingenspor, Thomas Klopstock, Holger Schulz, Eckhard Wolf, Wolfgang Wursta, Tammy Gillis, Hiroko Wakimoto, Jonathan Seidman, Marcy E. MacDonald, Susan Cotman, Valérie Gailus-Durner, Helmut Fuchs, Martin Hrabě de Angelis, Jong-Min Lee, Vanessa C. Wheeler

**Affiliations:** 1 German Mouse Clinic, Institute of Developmental Genetics, Helmholtz Zentrum München, Neuherberg/Munich, Germany; 2 Center for Human Genetic Research, Massachusetts General Hospital, Boston, Massachusetts, United States of America; 3 Comprehensive Pneumology Center, Institute of Lung Biology and Disease, Helmholtz Zentrum München, Neuherberg/Munich, Germany; 4 German Mouse Clinic, Institute of Experimental Genetics, Helmholtz Zentrum München, Neuherberg/Munich, Germany; 5 Department of Medicine III, Division of Cardiology, University of Heidelberg, Heidelberg, Germany; 6 Molecular Nutritional Medicine, Else Kröner-Fresenius Center and ZIEL Research Center for Nutrition and Food Sciences, Technische Universität München, Munich, Germany; 7 Department of Neurology, Friedrich-Baur-Institute and German Center for Vertigo and Balance Disorders, Ludwig-Maximilians-Universität München, Munich, Germany; 8 Institute of Epidemiology I, Helmholtz Zentrum München, Neuherberg/Munich, Germany; 9 Chair for Molecular Animal Breeding and Biotechnology, Gene Center, Ludwig-Maximilians-Universität München, Munich, Germany; 10 Max Planck Institute of Psychiatry, Munich, Germany; 11 Deutsches Zentrum für Neurodegenerative Erkrankungen, Standort München, Munich, Germany; 12 Department of Genetics, Harvard Medical School, Boston, Massachusetts, United States of America;; University of Florida, United States of America

## Abstract

Huntington’s disease (HD) is an autosomal dominant neurodegenerative disorder caused by the expansion of a CAG trinucleotide repeat in the *HTT* gene encoding huntingtin. The disease has an insidious course, typically progressing over 10-15 years until death. Currently there is no effective disease-modifying therapy. To better understand the HD pathogenic process we have developed genetic *HTT* CAG knock-in mouse models that accurately recapitulate the HD mutation in man. Here, we describe results of a broad, standardized phenotypic screen in 10-46 week old heterozygous *HdhQ111* knock-in mice, probing a wide range of physiological systems. The results of this screen revealed a number of behavioral abnormalities in *HdhQ111*/+ mice that include hypoactivity, decreased anxiety, motor learning and coordination deficits, and impaired olfactory discrimination. The screen also provided evidence supporting subtle cardiovascular, lung, and plasma metabolite alterations. Importantly, our results reveal that a single mutant *HTT* allele in the mouse is sufficient to elicit multiple phenotypic abnormalities, consistent with a dominant disease process in patients. These data provide a starting point for further investigation of several organ systems in HD, for the dissection of underlying pathogenic mechanisms and for the identification of reliable phenotypic endpoints for therapeutic testing.

## Introduction

Huntington’s disease (HD) is a dominantly inherited neurodegenerative disorder characterized by abnormal voluntary and involuntary movements, cognitive impairment and psychiatric disturbances. Onset typically occurs in mid-life, relentlessly leading to death after ~10-15 years [1]. While the brain is the primary site of pathology, with neurons in the striatum and cortex being particularly vulnerable, multiple non-neuronal abnormalities including weight loss and cardiac failure occur in the disease [2]. 

HD is caused by an expansion >35 repeats of a polymorphic CAG repeat tract within exon 1 of the *HTT* gene, elongating a glutamine stretch at the amino-terminus of an ~350 kDa protein called huntingtin [3]. Stringent genotype-phenotype analyses in HD patients reveal that the length of the expanded CAG repeat is the predominant factor determining the rate of the process that leads to onset of motor symptoms. Further, onset age is not influenced by either the normal CAG allele or by the presence of a second mutant allele, indicating that the longer expanded CAG allele determines motor onset in a fully dominant manner [4]. These data are consistent with the polyglutamine expansion conferring a novel property/properties on huntingtin, and/or enhancing a normal huntingtin function(s) such that the mechanism determining disease onset is saturated by a single dose of the mutant protein [5,6]. 

A number of genetic rodent models have been developed to investigate the pathogenesis of HD (see review by Heng et al. [7]). We have developed lines of *HTT* CAG knock-in mice (*HdhQ20*, *HdhQ50*, *HdhQ92*, *HdhQ111*) on various genetic backgrounds, in which different CAG repeat lengths are inserted into the mouse *HTT* homologue (*Htt* or *Hdh*) gene [8–10], accurately recapitulating human *HTT* alleles. Our long-term goal is to identify in knock-in mice CAG length-dependent phenotypes that are most proximal to the *HTT* CAG expansion mutation and that are caused by a single mutant *HTT* allele as in the majority of HD patients, as these are the most likely to provide relevant targets for therapeutic intervention. 

To date, several phenotypes have been identified in these mice, mostly in the *HdhQ92* and *HdhQ111* lines harboring the longest repeat tracts. These include nuclear huntingtin localization and inclusion phenotypes [10-12], somatic instability [9,10], elevated endoplasmic reticulum (ER) stress [13,14], altered cell signaling [15-17], impaired synaptic plasticity [18], behavioral abnormalities [12,19-25] and late stage brain pathology [12,26]. A number of these phenotypes were observed in heterozygous mice, varied with CAG length and displayed striatal selectivity, consistent with a dominant CAG length-dependent mechanism that determines disease onset in patients [11,14]. 

 The complexity of the disease and its multisystemic manifestations highlight the need for better, more global, unbiased and standardized phenotyping. Here, as part of our ongoing phenotyping effort, we report results from a broad, standardized phenotypic screen in heterozygous *HdhQ111* mice on a C57BL/6J genetic background, in collaboration with the German Mouse Clinic (GMC) (www.mouseclinic.de). We have thus identified a number of novel phenotypes in mice ranging from 10 to ~50 weeks of age that highlight neurological deficits but also indicate deficiencies in a number of other organ systems. The results of these analyses provide an important starting point for further exploration of underlying disease mechanisms and potential endpoints for testing of genetic or pharmacological modifiers. 

## Materials and Methods

### Mice

Mouse work was in accordance with the National Institutes of Health Guide for the Care and Use of Laboratory Animals. This study was reviewed and approved by the Massachusetts General Hospital (MGH) Subcommittee of Research Animal Care (SRAC), which serves as the Institutional Animal Care and Use Committee (IACUC) for MGH. The study was also reviewed and approved to be in accordance with German legal guidelines and by authority of the Regierung von Oberbayern.

Mice used in this study were the *HdhQ111* line [9] on a C57BL/6J genetic background [27]. Mouse genotyping and CAG repeat length determination were performed as described previously [28]. The mice used in the screen were heterozygous *HdhQ111*/+ mice and their wild-type Hdh+/+ littermates generated in crosses between male *HdhQ111*/+ C57BL/6J and female wild-type C57BL/6J mice (Jackson labs). All mice used in the screen were born within one week of each other and were separated based on sex but not genotype. At 5-6 weeks of age mice were shipped to the GMC and allowed to acclimatize to their new environment for four weeks before testing began at 11 weeks of age. Three pipelines of mice were sent to the GMC for phenotyping. Pipeline 1 comprised 20 *HdhQ111*/+ (10 males, CAG 117-129; 10 females, CAG 117-126) and 20 Hdh+/+ (10 males,10 females) mice. Pipeline 2 comprised 20 *HdhQ111*/+ (10 males, CAG 113-129; 10 females, CAG 117-126) and 21 Hdh+/+ (11 males, 10 females) mice and pipeline 3 comprised 20 *HdhQ111*/+ (10 males, CAG 113-129; 10 females, CAG 117-126) and 20 Hdh+/+ (10 males, 10 females) mice. Follow-up studies were performed at MGH using additional cohorts of *HdhQ111* C57BL/6J mice. Cohort 1: 19 *HdhQ111*/+ (10 males, CAG 124-128; 9 females, CAG 124-129) and 20 Hdh+/+ (10 males, 10 females); cohort 2: 20 *HdhQ111*/+ (10 males, CAG 121-128; 10 females, CAG 120-128) and 20 Hdh+/+ (10 males, 10 females); cohort 3: 19 *HdhQ111*/+ (10 males, CAG 124-132; 9 females, CAG 123-129 CAGs) and 18 Hdh+/+ (9 males, 9 females). 

### Phenotyping

The GMC screen (www.mouseclinic.de) comprises extensive, standardized phenotyping in the realms of dysmorphology, cardiovascular health, energy metabolism, clinical chemistry, eye, lung function, molecular phenotyping, behavior, neurology, nociception, immunology, steroid metabolism and pathology. The phenotypic tests that were part of the GMC screen are summarized in Table S1, and behavioral tests perfomed in MGH cohorts are summarized in Table S2. Note that not all animals in each GMC pipeline were tested in each paradigm; the specific numbers that relate to the data are indicated in the figure legends. Phenotyping screens at the GMC were performed according to standardized methods described in [29–31]. Detailed methods for the results described in this manuscript are also provided in a Methods S1 file. 

### Statistical analyses

Data were generally analyzed either by 2-way ANOVA (genotype, sex and genotype x sex interaction as variables) or independently in males and females using 2-tailed unpaired Student’s t-tests. Depending on the nature of the test, GMC typically analyzes their screen data using one or other of these statistical tests. Rotarod learning data that incorporated time as a variable was also analyzed using linear mixed models that included day of testing and a day x genotype interaction term. Where we have observed a significant genotype effect in ANOVA we have performed Sidak correction for multiple comparisons (mutant versus wild-type for males and mutant versus wild-type for females). We report nominal p values in the text, unless specifically stated otherwise. Graphs are annotated with asterisks to indicate the sex-stratified p values using t-tests, or the sex-stratified p values following Sidak’s multiple testing correction. Results throughout are displayed as mean±standard error (SEM). A p-value <0.05 was used as the level of signiﬁcance. This study is designed to be a hypothesis-generating screen. We have therefore reported both results with nominally significant p values, as well as those that do not reach statistical significance but which may be of interest in the context of other data presented here or elsewhere. 

## Results

Table 1 summarizes the phenotypes that are described below in *HdhQ111*/+ mice. 

**Table 1:** Summary of *HdhQ111*/+ phenotypes.

**Age (wk)**	**Test**	**Phenotype**
11	Open field (light)	Increased time in center; increased time resting in center
	Dark/light box	*(Increased number of entries)*
12	Rotarod	Increased motor learning
	Cardiovascular parameters	Increased heart rate
14-16	Social discrimination	Impaired in males
16	DEXA	aBone mineral density increased in females, decreased in males; (*decreased fat content*)
16-19	Clinical chemistry	Decreased glucose in free-fed mice; (*decreased cholesterol in free-fed mice*)
18	Lung function	Increased tidal volume
19	Cardiovascular parameters	aIncreased heart weight/tibia length in females, decreased in males
24	Rotarod	Decreased motor learning
b28-36	Olfaction	Impaired olfactory discrimination
c40	Open field (dark)	Increased time in center; decreased distance covered; decreased velocity; decreased ambulatory time; ddecreased stereotypy; ddecreased vertical time
46	Catwalk	Decreased stride length; decreased cruciate step pattern
	Vertical pole descent	Increased time to turn and descend
	Weight	Decreased in females; (*decreased in males*)

Standard font indicates nominally significant p-values (p<0.05); italic font with parentheses indicates a trend with p-values >0.05. See text for details.

^a^ Significant genotype x sex interaction;

^b^ Olfactory discrimination deficit also observed in male mice at 24-27 weeks and 42-47 weeks and in female mice at 50-56 weeks.

^c^ Open field testing in the dark in a separate cohort at 56-59 weeks showed same phenotypes as cohort at 40 weeks with the exception that the 56-59 week mice did not spend more time in the center of the field or show a difference in vertical activity.

^d^ Noted in periphery of open field only.

### General phenotypic observations

A whole body visual assessment of *HdhQ111*/+ mice and their wild-type littermates, carried out at 10 weeks of age, revealed no abnormalities in body appearance, craniofacial/head morphology, limbs, digits, tail, eye, ears, teeth, vibrissae, coat, hair follicles, skin pigmentation/condition, muscle morphology, seizures, motor capabilities/coordination, movement, eating/drinking behavior, respiratory system, or reproductive/urinary system. Dual energy X-ray absorption (DEXA) analyses at 16 weeks of age also revealed the absence of any bone morphological abnormalities with the exception of a slight increase in bone mineral density in females and decrease in males (females: Hdh+/+ (N=10) 46±1 mg/cm^2^, *HdhQ111*/+ (N=10) 49±1 mg/cm^2^; males: *Hdh+/+* (N=10) 49±1 mg/cm^2^, *HdhQ111*/+ (N=10) 46±1 mg/cm^2^; ANOVA sex x genotype interaction p<0.05). No hearing (10 weeks) or eye abnormalities (15, 17 weeks) were detected. Macroscopic assessment and a broad histological examination by hematoxylin and eosin staining at 19 weeks of age did not reveal overt pathology in any tissue. Together these observations support previous findings that huntingtin’s critical role in development is not compromised by the CAG repeat expansion [8], and are consistent with a single expanded CAG allele eliciting subtle gain of function phenotypes and an insidious disease course. 

### Analyses of neurological dysfunction

To probe neurological dysfunction *HdhQ111*/+ mice underwent a series of behavioral and neurological tests (Table S1). *HdhQ111*/+ mice did not show any abnormalities in grip strength or in the modified SHIRPA test at 11 weeks (data not shown). Analyses of acoustic startle and pre-pulse inhibition at 13 weeks indicated very minor, though not statistically significant, reductions in acoustic startle response but no alterations in pre-pulse inhibition (Figure S1). Behavioral phenotypes identified are described in subsequent sections.

### *HdhQ111/*+ mice display abnormal behavioral responses to a novel environment

**Figure 1:**
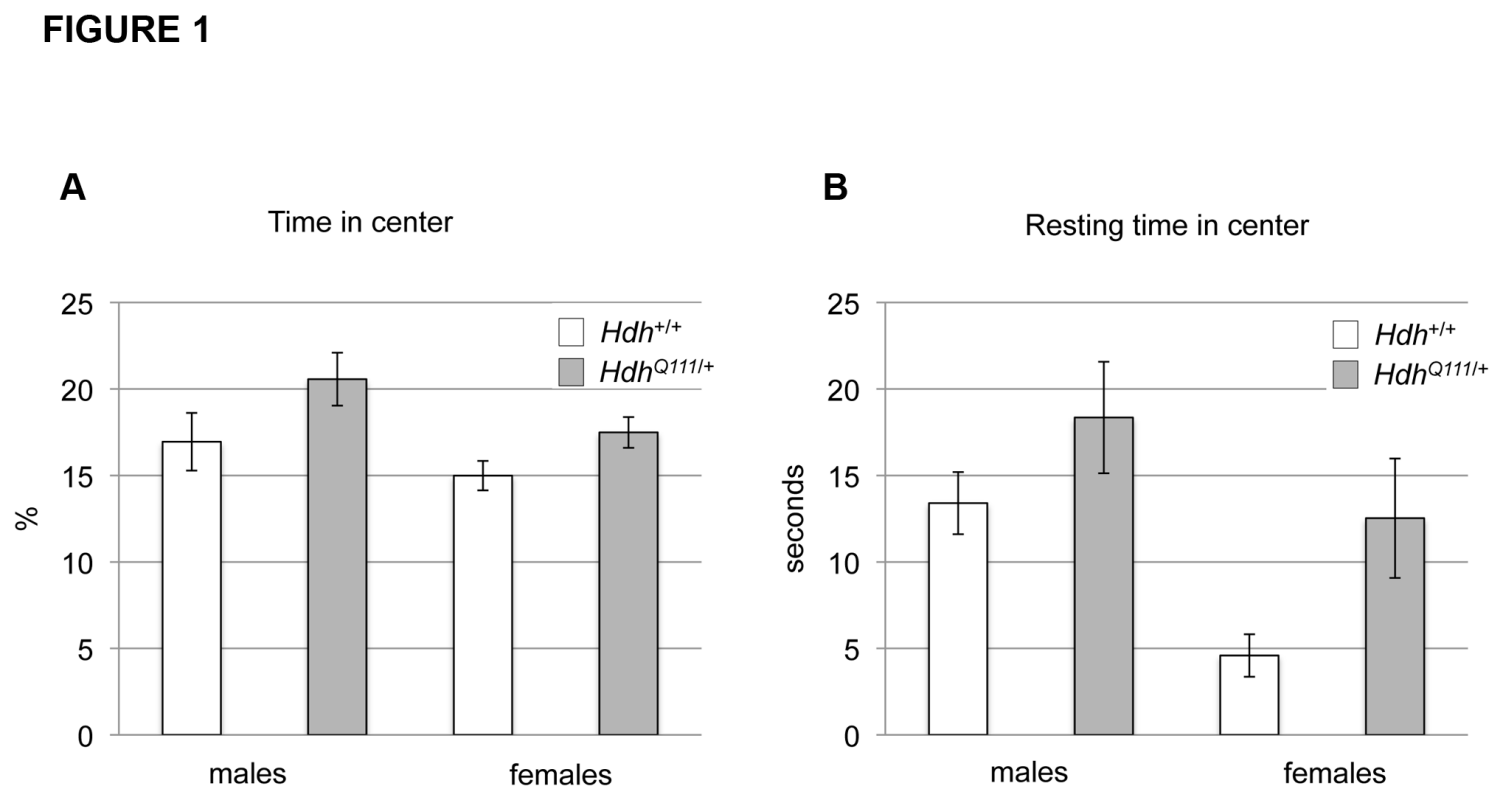
*HdhQ111*/+ mice display reduced anxiety-like behavior in the open field. *HdhQ111*/+ and wild-type littermates were tested in the open field arena at 11 weeks of age during the light phase of the diurnal cycle. *HdhQ111*/+ mice were found to spend more time in the center of the open field arena (A), and when in the center, spent more time resting (B). Y-axis in (A) shows the percentage of time over the 20-minute testing period spent in the center, and in (B) the total time resting in the center in the 20-minute testing period. N=20 *HdhQ111*/+ mice (10 males, 10 females) and N=21 Hdh+/+ mice (11 males, 10 females). Bars show mean±SEM. ANOVA genotype effect p<0.05 for both (A) and (B).

Behavioral response to a novel environment was tested in the open field arena in the light phase of the diurnal cycle at 11 weeks of age. *HdhQ111*/+ mice did not show any difference in distance traveled, speed of travel or rearing activity (data not shown), revealing the lack of significant alteration of exploratory or locomotor activity in this paradigm. However, *HdhQ111*/+ mice were found to spend more time in the center of the open field than their wild-type littermates (ANOVA genotype effect p<0.05) (Figure 1A) and tended to rest in the center for longer periods (ANOVA genotype effect p<0.05) (Figure 1B). As mice tend to be averse to open spaces, these findings suggested that *HdhQ111*/+ mice exhibited decreased anxiety-like behavior. Repeated measures analyses did not reveal any significant differences in habituation to the novel environment over time for any measure (data not shown). 

As this was a very early time-point relative to the onset age for other reported behavioral phenotypes in these mice we were interested to test the reproducibility of this finding across different laboratories/environments by testing cohorts of mice in a similar open field paradigm in follow-up studies at MGH. Note that the open field apparatus differed between the two institutions (Materials and Methods). We were able to confirm the open field phenotypes of increased time in the center in one cohort (MGH cohort 1, mean age 9.6 weeks: males Hdh+/+ 280±20s vs. *HdhQ111*/+ 303±17s; females Hdh+/+ 253±12s *vs HdhQ111*/+ 339±25s; ANOVA genotype effect p<0.01), in female mice of a second cohort (MGH cohort 3, mean age 10.8 weeks: males Hdh+/+ 278±14s vs. *HdhQ111*/+ 199±13s; females Hdh+/+ 226±20s *vs HdhQ111*/+ 301±16s; ANOVA genotype-sex interaction effect p<0.0005) but not in a third cohort (MGH cohort 2, mean age 9.25 weeks: males Hdh+/+ 277±19s vs. *HdhQ111*/+ 273±25s; females Hdh+/+ 292±16s *vs HdhQ111*/+ 285±17s; ANOVA genotype effect p=0.80). Taken together our results suggest that *HdhQ111*/+ mice do indeed display open field phenotypes as early as approximately 10 weeks of age that are robust enough to withstand differences between mouse testing facilities/apparatus. However, these phenotypes are not fully penetrant at this age, and appear more pronounced in females than in males. 

An additional test of anxiety using a dark-light box at 11 weeks of age was performed on a separate cohort of mice at the GMC (pipeline 3, see Table S1). In the dark-light box, *HdhQ111*/+ mice showed a trend towards an increased number of light box entries (males: Hdh+/+ 13.8±1.0, *HdhQ111*/+ 15.7±1.4; females: *Hdh+/+* 1.57±0.97, *HdhQ111/+* 18.8±1.62; ANOVA genotype effect p=0.08), indicating reduced anxiety compared to wild-type mice and consistent with findings in the open field arena. Overall, the results suggest that young *HdhQ111*/+ mice display behavior consistent with decreased anxiety. 

### *HdhQ111/*+ mice are hypoactive in the dark phase

**Figure 2:**
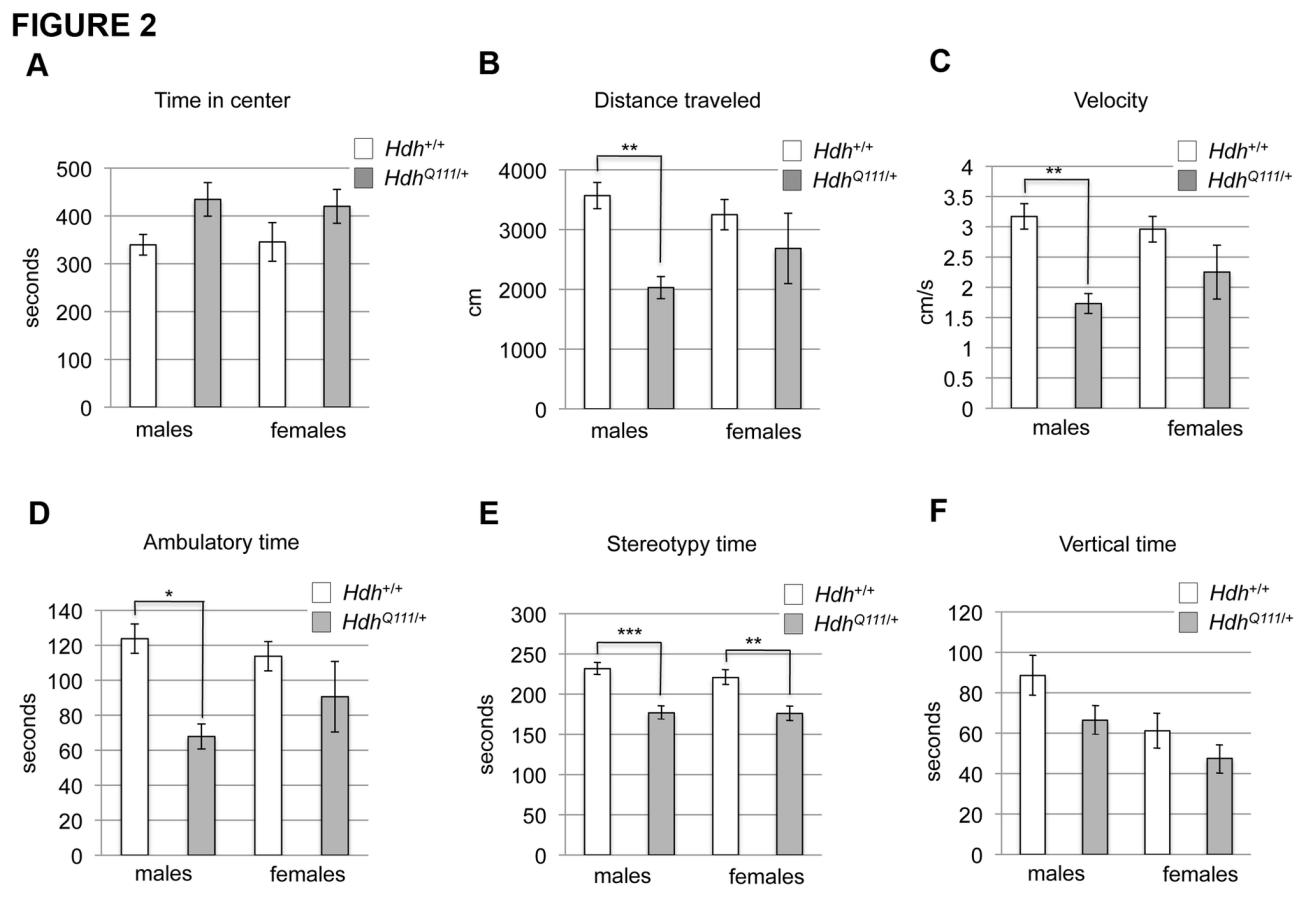
*HdhQ111*/+ mice exhibit hypoactivity in the open field. *HdhQ111*/+ and wild-type littermates were tested in the open field arena at 40 weeks of age during the dark phase of the diurnal cycle. Consistent with observations in younger mice, *HdhQ111*/+ mice spent more time in the center of the open field (A). *HdhQ111*/+ mice also exhibited a decrease in distance traveled (B), velocity (C) and in time spent in ambulatory motion (D) in the whole testing arena, indicating that they were hypoactive. (E) and (F) show time spent in stereotypic movements or rearing, respectively, in the periphery of the open field. All measurements are over the 20-minute testing period. N=19 *HdhQ111*/+ mice (10 males, 9 females) and 20 Hdh+/+ mice (10 males, 10 females). Bars show mean±SEM. All tests showed a significant genotype p value in a 2-way ANOVA (see text for p values). Asterisks indicate adjusted p values following multiple testing correction. * p<0.05; **p<0.01; ***p<0.001.

Given the above findings we were interested to use the open field arena to examine mouse activity during the dark (active) phase. We performed open field testing on a cohort of mice at MGH (MGH cohort 1) at 40 weeks of age in the dark phase. The results of this test are displayed in Figure 2. *HdhQ111*/+ mice spent more time in the center of the open field (Figure 2A: ANOVA genotype effect p<0.02), covered less distance in the open field arena (Figure 2B: ANOVA genotype effect p<0.005), moved at a decreased velocity (Figure 2C: ANOVA genotype effect p<0.001) and spent less ambulatory time (Figure 2D: ANOVA genotype effect p<0.005) than their wild-type littermates. All of these effects were seen in both the center and periphery of the open field (data not shown separately for center and periphery). Significantly reduced stereotypy time (Figure 2E: ANOVA genotype effect p<0.001) and vertical time Figure 2F: ANOVA genotype effect p<0.05) were also noted in the periphery of the open field. These results suggest that by 40 weeks of age *HdhQ111*/+ mice are hypoactive and display a measure of decreased anxiety that was already observed at 10-11 weeks of age. Open field analysis in the dark phase was also carried out in an independent cohort of mice (MGH cohort 3) at 56-59 weeks of age (Table S3). The 56-59 week cohort displayed many of the same phenotypes as the 40 week cohort, notably reduced distance travelled, reduced velocity, reduced ambulatory time and reduced stereotypy time in the periphery. Time in the center of the open field was not significantly different, though we note that the 56-59 week old mice overall spent less time in the center than the 40 week mice (Figure 2, Table S3). Overall, there was a high degree of reproducibility between the 40- and 56-59- week cohorts, with hypoactivity being a notable common feature. 

Further longitudinal open field testing under both light and dark paradigms will be needed to better assess the timeframe within which these behavioral phenotypes become manifest and their potentially progressive nature.

### *HdhQ111/*+ mice exhibit motor learning and coordination deficits

**Figure 3:**
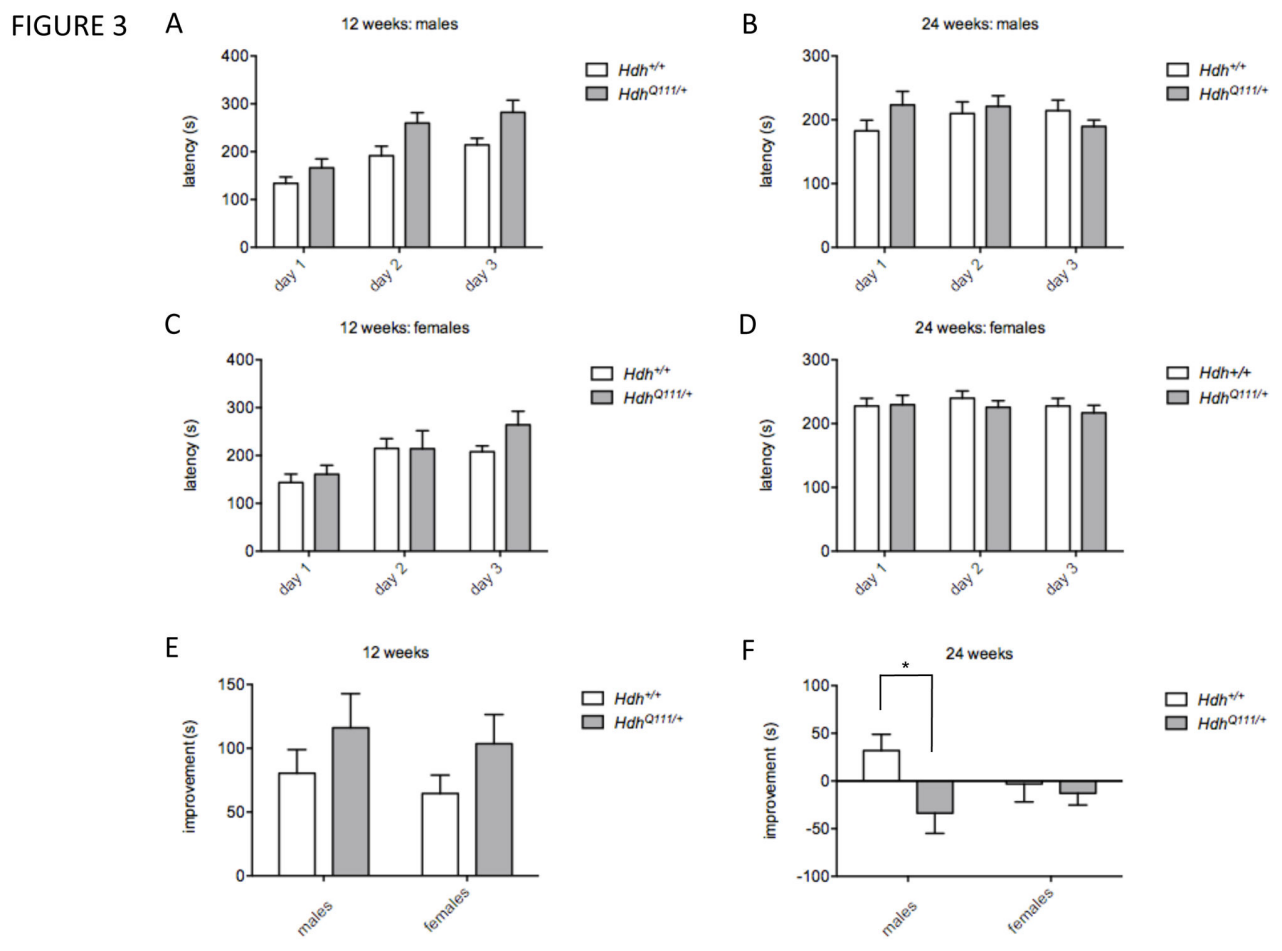
Performance on the accelerating rotarod. *HdhQ111*/+ and wild-type littermates were tested at 12 weeks (A, C, E) and 24 weeks (B, D, F) on an accelerating rotarod over three consecutive days and latency to fall (seconds, s) was recorded on each day (A-D). Improvement (E, F) was measured as the difference in latency to fall on day 3 compared to day 1, with positive values indicating improvement and negative values indicating a worsening performance. 12 weeks: N=20 *HdhQ111*/+ mice (10 males, 10 females and 21 Hdh+/+ mice (11 males, 10 females); 24 weeks: N= 20 *HdhQ111*/+ mice (10 males, 10 females) and 20 Hdh+/+ mice (10 males, 10 females). Bars show mean±SEM. * Adjusted p value (p<0.05) following multiple testing correction.

Tests of motor function in the GMC screen comprised the accelerating rotarod, gait analysis and vertical pole descent. Rotarod analysis was carried out at 12 weeks of age. As assessed in a single day trial, *HdhQ111*/+ mice did not show any altered latency to fall off the rotarod. However, in a three-day trial paradigm to assess motor learning, *HdhQ111*/+ mice showed increased latencies compared to Hdh+/+ mice (linear mixed effects model, genotype effect p<0.05) (Figure 3A,C). Learning over test days was somewhat increased in mutants (Figure 3A,C,E) but missed statistical significance when analyzed by including a genotype x day interaction term in the model (linear mixed effects model, genotype x day p=0.097). To explore this phenotype further, a cohort of mice was tested in the same rotarod paradigm at MGH at 10 weeks of age and again at 24 weeks of age. At 10 weeks of age *HdhQ111*/+ did not show significant genotype differences in latency to fall or learning, consistent with the slightly younger age of the MGH cohort compared to the GMC cohort (data not shown). At 24 weeks of age we did not find that mutant and wild-type mice differed significantly in latency to fall (linear mixed effects model, genotype effect p=0.83) (Figure 3B, D). However, we found that many mice, both mutant and wild-type, showed a worsened, rather than an improved performance, over the 3-day trial (Figure 3B, D, F). Interestingly however, this effect was more pronounced in *HdhQ111*/+ mice than in wild-type controls (linear mixed effects model, genotype x day p=0.024). Performing a 2-way ANOVA using “improvement” as the dependent variable followed by a Sidak multiple correction test indicated that the worsened performance on the rotarod of 24 week *HdhQ111*/+ mice was largely driven by an effect in the males (Figure 3F) (ANOVA genotype effect p<0.05; corrected p value in males =0.029). It appears that under this experimental paradigm the 24-week old mice may not have had sufficient time to train on the rotarod in order to improve their performance. However, these conditions appear to have inadvertently exposed a motor learning deficit in mutant mice. Note that improvement (or worsening) in performance over 3 days was not influenced by mouse weight (data not shown). 

**Figure 4:**
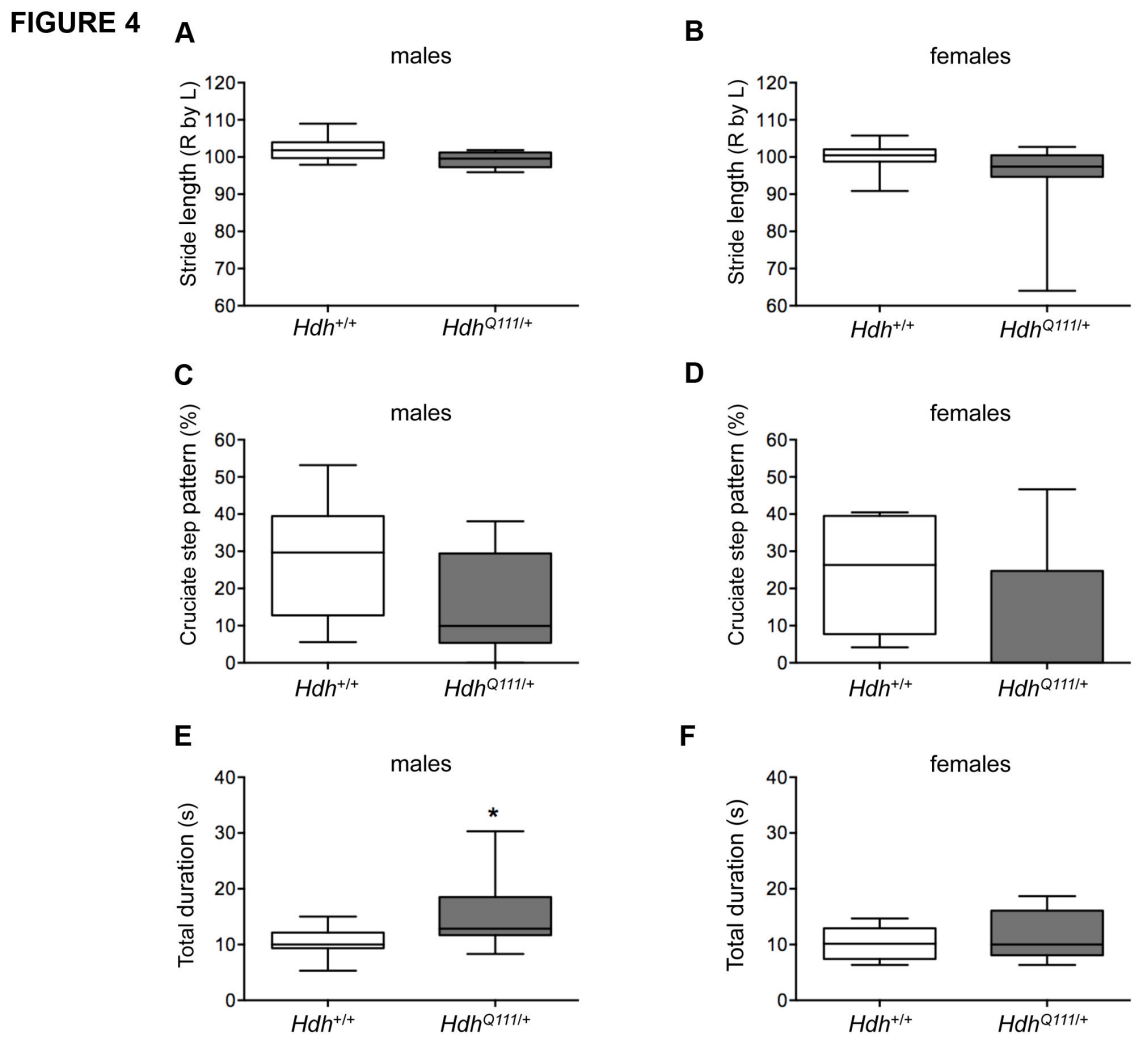
Motor deficits in aged *HdhQ111*/+ mice. *HdhQ111*/+ and Hdh+/+ mice at 46 weeks of age were tested on an automated CatWalk system (A-D) and for their ability to descend a vertical pole (E,F). *HdhQ111*/+ mice showed reduced Right by Left hindlimb stride length (A,B), a decreased cruciate step pattern frequency of pattern left front-right front-left hind-right hind (C,D) and an increased time to descend a vertical pole (total duration being the time taken to turn at the top of the pole and descend to the bottom). . For the CatWalk N=10 per sex and genotype; for the pole descent, males N=8 Hdh+/+, N=9 *HdhQ111*/+, females N=10 per genotype. Plotted are Tukey box-whisker graphs. All tests showed a significant genotype p value in a 2-way ANOVA. * Adjusted p value (p<0.05) following multiple testing correction.

Gait analysis in 46-week old mice revealed that *HdhQ111*/+ mice exhibited a reduced stride length, mainly seen in males (ANOVA genotype effect p<0.05) (Figure 4A,B), and consistent with a reduced stride length observed in CD1.*HdhQ111* mice [12]. In addition, analyses of the step pattern sequence revealed that *HdhQ111*/+ mice used the more advanced cruciate step pattern less than the wild-type controls (ANOVA genotype effect p<0.05) (Figure 4C,D). The same mice were also tested at 46 weeks of age for their ability to descend a vertical pole. *HdhQ111*/+ mice needed more time than the wild type controls to turn around and to descend (ANOVA genotype effect p<0.05) (Figure 4, E.F). 

Together, these data indicate a biphasic response in *HdhQ111*/+ mice, with evidence of improved motor learning at an early age, followed by impaired motor learning and motor performance as the mice age. 

### *HdhQ111/*+ mice show impaired social discrimination

**Figure 5:**
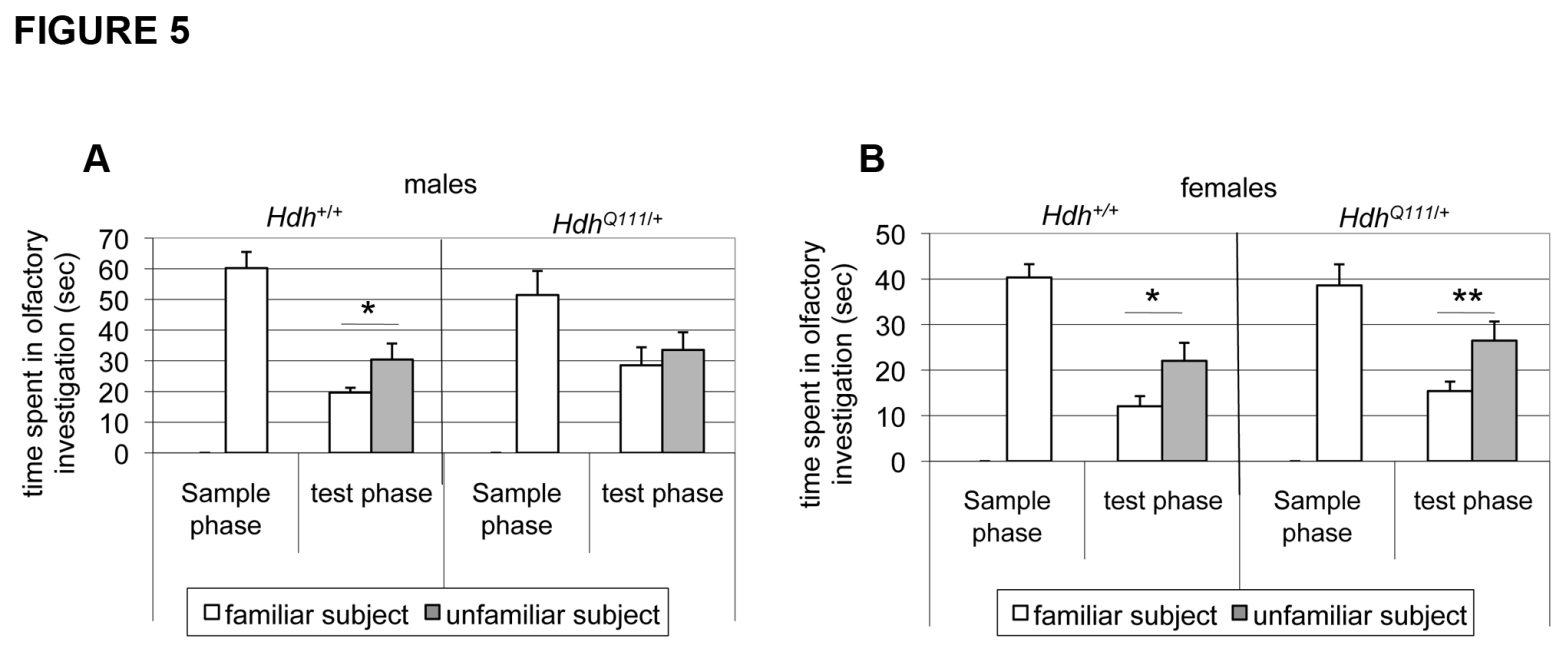
Male *HdhQ111*/+ mice display a social recognition deficit. In the sample phase, an ovariectomized (OVX) female mouse was introduced into a cage with a male (A) or female (B) experimental *HdhQ111*/+ or Hdh+/+ mouse and time spent in olfactory investigation was recorded for 4 minutes after which time the OVX female was removed. After two hours, in the test phase, the experimental mouse was returned to the testing cage with both the familiar OVX mouse and a novel unfamiliar OVX female and time spent in olfactory investigation with both the familiar and unfamiliar mouse recorded for 4 minutes. Males: N=10 per genotype; females: N=10 per genotype. Bars show mean±SEM. * p<0.05; ** p<0.01 (2-tailed unpaired Student’s t-test comparing time spent investigating familiar and unfamiliar mouse).

Recognition memory, the ability to discriminate familiar and unfamiliar stimuli, is impaired in HD [32]. To probe recognition memory in *HdhQ111*/+ mice we employed a social discrimination task that takes advantage of the innate drive to investigate an unfamiliar compared to a familiar mouse. After initial exposure to a test mouse the experimental subject acquires the olfactory signature of that mouse. Following a defined interval of time the experimental subject is reintroduced to the test mouse together with an unfamiliar mouse. The significantly longer time spent investigating the unfamiliar mouse reflects recognition of the original test mouse. Mice were tested at 14-16 weeks of age in this paradigm for their ability to discriminate between a familiar and an unfamiliar mouse. While wild-type males spent significantly more time investigating an unfamiliar mouse than a familiar mouse, male *HdhQ111/+* mice failed to display this discriminatory ability (Figure 5A). In contrast female *HdhQ111* mice did not exhibit this deficit, but showed comparable discriminatory abilities to their wild-type littermates (Figure 5B). The failure to discriminate between familiar and unfamiliar mice suggests that male *HdhQ111/+* mice may have impaired recognition memory. 

### *HdhQ111/*+ mice are impaired in their ability to discriminate odors

**Figure 6:**
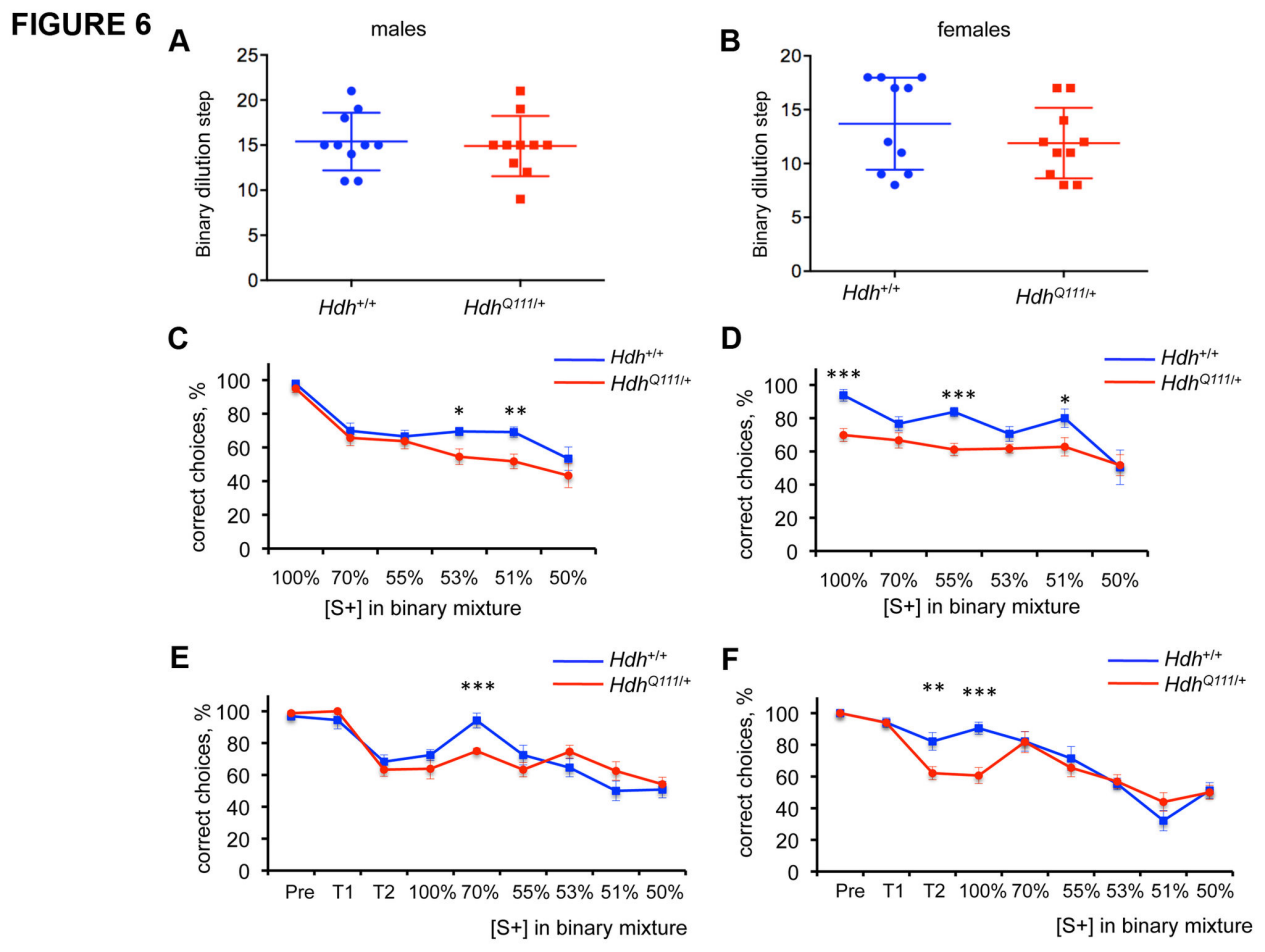
*HdhQ111*/+ mice exhibit an olfactory discrimination deficit. A,B: olfactory sensitivity was determined by testing the ability of the mice to recognize binary dilutions of the scent on which they had been trained. Plotted on the y-axis is the number of binary dilutions at which a particular mouse is able to recognize the scent. C-F: olfactory discrimination test. Mice were trained on a scent [S+] (either strawberry or apple) and then tested for their ability to recognize decreasing proportions of this scent in a binary mixture. C,D: test carried out at the GMC on mice at 28-36 weeks of age. N=10 per sex and genotype. E,F: test carried out at MGH on males at 42-47 weeks of age and females 50-56 weeks of age. Males Hdh+/+ N=9, *HdhQ111*/+ N=10; females Hdh+/+ N=9, *HdhQ111*/+ N=9). Pre: pre-training; T1: training step 1; T2: training step 2 (see Materials and Methods). Error bars show SEM. * p<0.05; ** p≤0.01, ***p<0.005 in 2-tailed unpaired Student’s t-test comparing mutant and wild-type mice at each dilution.

Premanifest HD mutation carriers exhibit olfactory deficits [33-38]. However, olfaction has not previously been reported at the functional level in a mouse model of HD. Given the early detection of this deficit in HD mutation carriers, and the highly developed sense of smell in the mouse, we took advantage of olfactory tests that had been developed by the GMC in our *HdhQ111*/+ screen [39]. These tests were performed between 28 and 36 weeks of age. Mice were trained to recognize one of two odors (strawberry or apple) and then tested both for their sensitivity to recognize the odor on which they had been trained following serial binary dilutions, and for their ability to discriminate the odor on which they had been trained when mixed with the alternative odor. Mutant mice showed no difference in olfactory sensitivity (Figure 6A,B). However, both male and female mutant mice displayed a clear deficit in their ability to discriminate between binary mixtures of the two odors (Figure 6C,D). Compared to male Hdh+/+ mice, male *HdhQ111*/+ mice showed an impairment in their ability to choose the correct odor when it comprised 53% (t-test p=0.04) or 51% (t-test p=0.01) of the mixture. Female *HdhQ111*/+ mice appeared more severely impaired in this task; they showed a significant difference compared to female Hdh+/+ mice in odor discrimination at mixtures of 55% (t-test p=0.0001) and 51% (t-test p=0.04). Further, even at 100% of the trained odor (ie. no mixture), female mutants showed a difference compared to wild-type mice in their ability to make the correct choice (t-test p=0.0002). 

 The olfactory discrimination test was repeated at MGH in male mice of 24-27 weeks of age (MGH pipeline 1) and in a separate group of male and female mice (MGH pipeline 3) at older ages (males 42-47 weeks; females 50-56 weeks). At 24-27 weeks of age, male *HdhQ111*/+ mice showed an impairment in their ability to discriminate between the two odors in the 70% (t-test p=0.007) and 53% (t-test p=0.03) mixtures (Figure S2). As we had observed genotype differences in odor recognition prior to the mixing steps (see “100%” on graphs in Figure 6D and Figure S2) we explored further a potential training deficit in *HdhQ111*/+ mice in our testing of the older MGH cohort by evaluating the number of correct/incorrect choices during training (see Materials and Methods for details of training steps) in addition to documenting the number of correct/incorrect choices upon mixing of the odors (Figure 6E,F). Male *HdhQ111*/+ mice exhibited a discrimination deficit at the 70% dilution step (t-test p=0.001) (Figure 6E), while female *HdhQ111*/+ mice exhibited a training deficit, apparent at training step 2 (t-test p=0.0095) and the 100% odor step (t-test p=0.0006) but did not show any significant difference in subsequent odor discrimination (Figure 6F). The more pronounced training deficit in females is consistent with the original GMC findings (compare “100%” in graphs of Figure 6C and D). Notably, we did not observe any genotype differences in training step 1, in which the mice are trained to look for the chocolate reward, but only in subsequent steps in which mice are trained to *associate* the conditioned odor with the reward. This argues against a perceptual deficit in the mutant mice but indicates a possible olfactory learning deficit. It is not surprising that there are differences in the precise mixtures at which a deficit is seen in mutants in the GMC and MGH studies as there are many factors that are extremely difficult to control, even in the same facility, which could influence the results. Differences may also be due in part to an age-related decline in the ability of the wild-type mice to discriminate odors (compare wild-type mice at 28-36 weeks in Figures 6C and D that maintain % correct choice across dilutions with wild-type mice at 42-47 weeks in Figure 6E and at 50-56 weeks in Figure 6F that show a decline in % correct choice across dilutions). These caveats notwithstanding, our data indicate the presence of an olfactory impairment in *HdhQ111*/+ mice that may at least in part result from an olfactory learning deficit. 

 Olfactory memory was also tested in the GMC cohort by testing the ability of the mice to recognize the odor on which they had been trained after an interval of 16-17 weeks or 20-22 weeks following the end of the initial olfactory discrimination test (Figure S3). While wild-type mice were still able to recognize the correct odor after 20-22 weeks, female *HdhQ111*/+ mice displayed a marked inability to do so after this time interval (20 week females: chi squared test p=0.0016). These findings support an olfactory learning and memory deficit that is more pronounced in female than male heterozygous mutant mice. 

### Metabolic assessment of *HdhQ111/*+ mice

**Figure 7:**
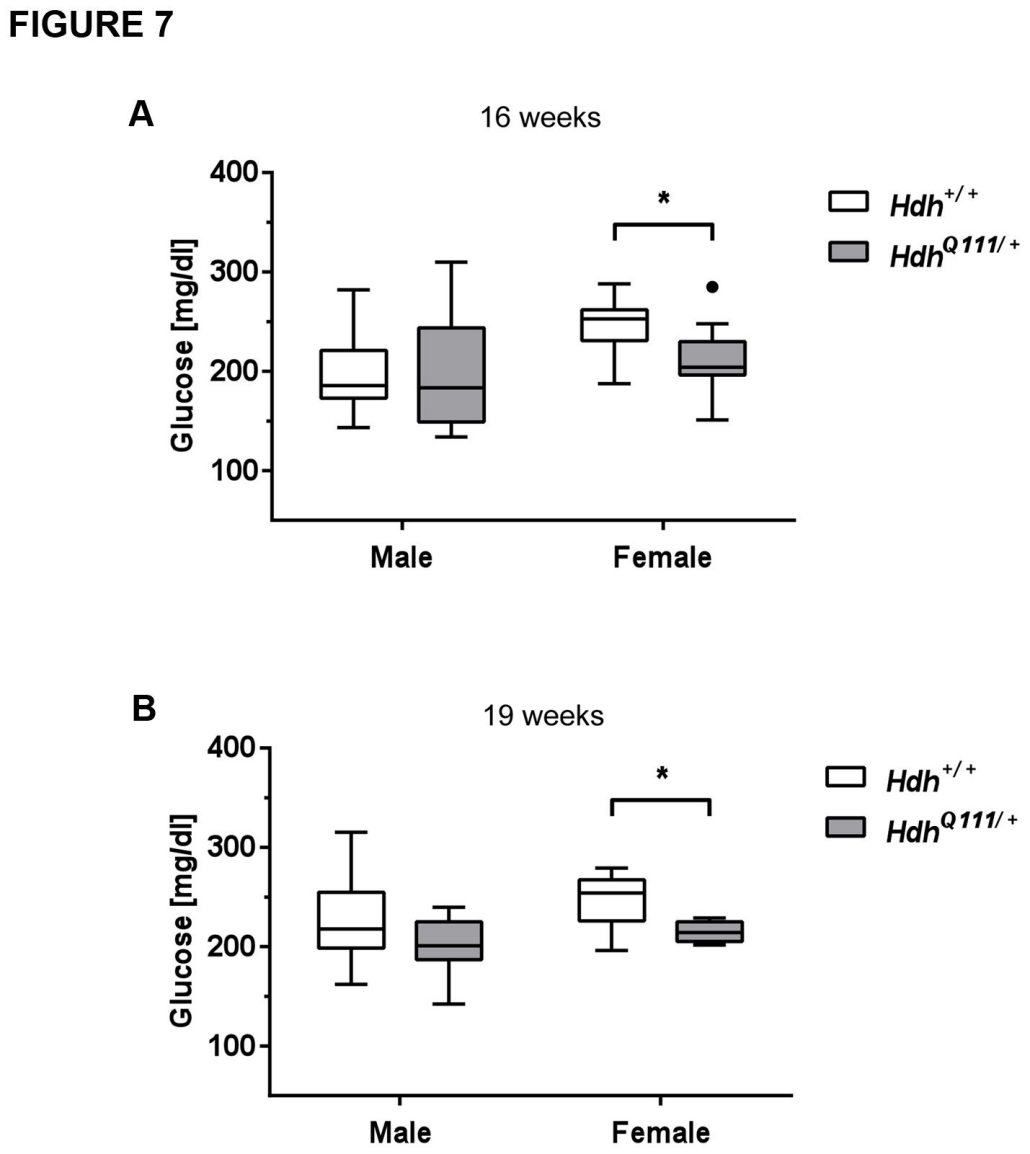
Free fed glucose levels in *HdhQ111*/+ and Hdh+/+ mice. Glucose levels were measured in free-fed mice at 16 weeks of age (A) (N=10 for each sex and genotype) and again at 19 weeks (B) in the same cohort of mice (N=10 males for each genotype, N=9 Hdh+/+ females and N=7 *HdhQ111*/+ females). * p<0.05 (2-tailed unpaired Student’s t-test). Plotted are Tukey box-whisker graphs.

With increasing indications that HD can be considered a multisystemic disease we were interested in peripheral phenotypes in *HdhQ111* mice. There is considerable evidence for an energy deficit in HD. Patients exhibit progressive weight loss that appears to stem from an increased metabolic rate [40,41]. Body weight was measured in three cohorts of mice at five time points from 11-46 weeks of age. No genotype differences in body weight were found in mice up to 19 weeks of age (Table S4) although interestingly, there was a trend towards a lower fat content in *HdhQ111*/+ mice at 16 weeks of age (males Hdh+/+ 10.68±2.00% vs. *HdhQ111*/+ 8.82±1.47%; females Hdh+/+ 6.57±1.95% vs. *HdhQ111*/+ 4.51±1.53%). By 46 weeks of age mutant mice exhibited a slight weight decrease compared to their wild-type littermates (Table S4: t-test: males p=0.09; females p<0.05). Consistent with the comparable weights between mutant and wild-type mice up to 19 weeks of age indirect calorimetry did not identify alterations in measures relating to energy uptake and expenditure (mean O_2_ consumption rate, respiratory exchange ratio, heat production, food consumption, rectal body temperature or activity) at 13 weeks of age (data not shown). HD patients have also been found to exhibit altered glucose homeostasis, including a higher incidence of diabetes in some studies [42-44]. We did not find evidence of glucose intolerance in *HdhQ111*/+ mice as determined by an intraperitoneal glucose tolerance test (IpGTT) at 14 weeks (Figure S4). We noted that in the IpGTT blood peak glucose levels, particularly in female *HdhQ111*/+ mice, were consistently very slightly lower than those of wild-type mice, although this difference was not statistically significant. (Figure S4). Consistent with this, an independent cohort of female *HdhQ111*/+ mice at 16-19 weeks of age showed significantly (p<0.05) lower blood glucose levels in a fed state (Figure 7, Table S5), though fasting glucose levels were not altered (Table S5). Note that other blood metabolites, proteins, enzyme activities and electrolytes, measured at 16-19 weeks, were largely unaltered (Table S5). Of interest, cholesterol levels showed a trend towards reduced levels in free-fed *HdhQ111*/+ mice (Table S5) consistent with altered cholesterol metabolism previously reported in patients and mice [45]. Taken together, these results indicate that up to approximately 19 weeks of age, a single mutant *HTT* allele does not grossly disrupt homeostasis in the mice. However, subtle alterations in young *HdhQ111*/+ mice, together with weight loss in older mice, are suggestive of ongoing metabolic disturbances that warrant further investigation. 

### Cardiovascular and lung abnormalities in *HdhQ111/*+ mice suggest autonomic nervous system defects

**Figure 8:**
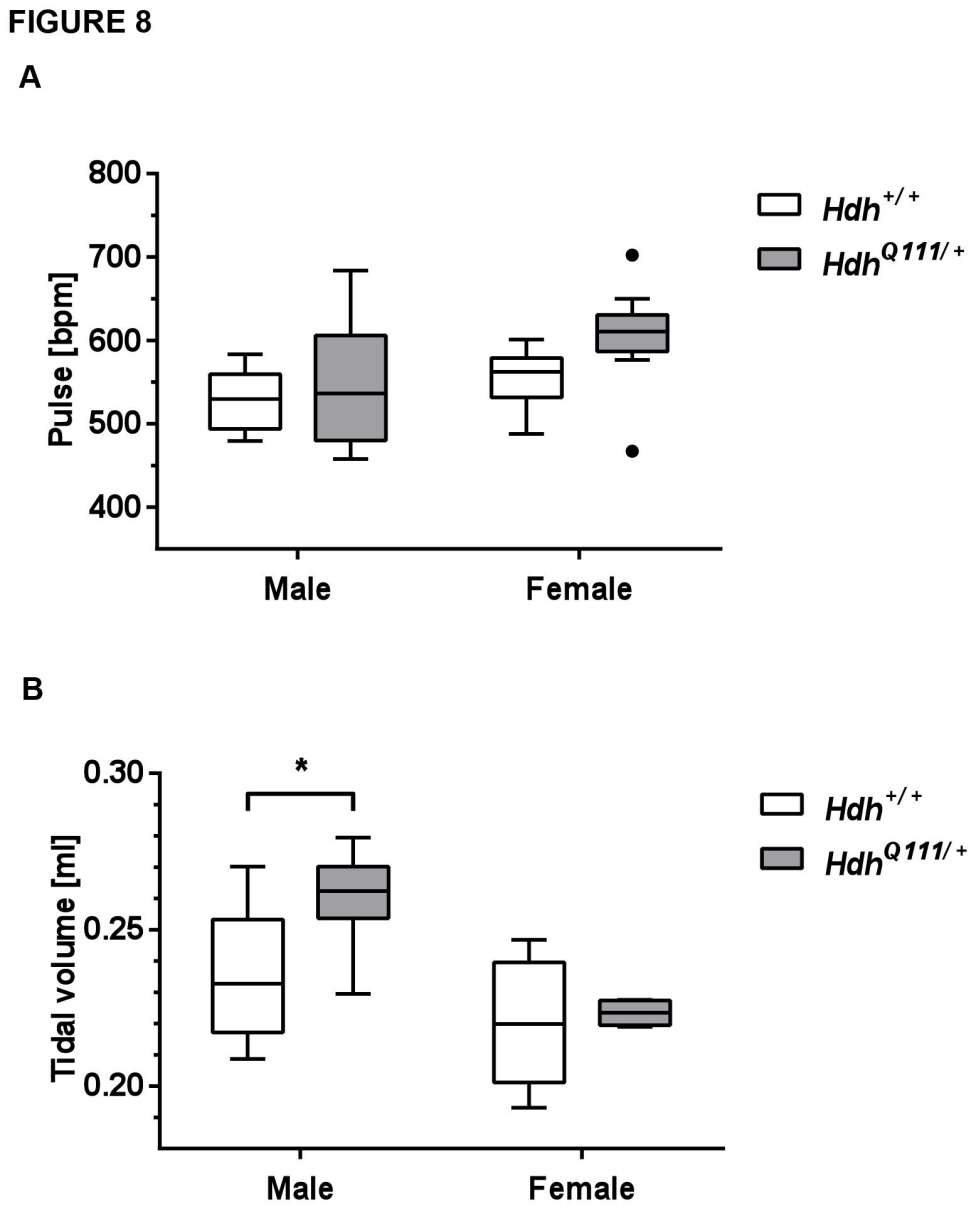
Altered cardiovascular and lung parameters in *HdhQ111*/+ mice. A. Pulse was measured in 12 week mice (N=10 per group). A significant genotype p value (p<0.05) was obtained in a 2-way ANOVA. B. Tidal volume was measured in an independent cohort of 18 week mice (n=6 per group). Tidal volume and other lung function measures were analyzed independently in males and females by 2-tailed unpaired student’s t-test. * p<0.05. Plotted are Tukey box-whisker graphs.

Blood pressure parameters were determined at 12 weeks of age (GMC pipeline 1; see Table S1). No differences were found in systolic, diastolic or mean arterial pressure compared between *HdhQ111*/+ and *Hdh+/+* mice (Table S6). However, *HdhQ111*/+ mice were found to have an increased heart rate compared to Hdh+/+ mice (ANOVA genotype effect p<0.05; Figure 8A, Table S6). Additional measures of heart disease (heart weight, proatrial natriuretic peptide (Nt proANP)) were made in a separate cohort of mice (GMC pipeline 2; see Table S1) at 19 weeks of age (Table S6). There were no genotype differences in Nt proANP levels, however we identified a sex-genotype interaction (ANOVA interaction effect p<0.05) for heart weight normalized to tibia length, with female mutants exhibiting a slightly increased heart weight/tibia length and male mutants exhibiting a slightly lower heart weight/tibia length compared to wild-type mice (Table S6). However, the effect is small, and together with the lack of any change in Nt proANP we conclude that there are no obvious signs of heart disease in these mice. Cardiac function was also assessed in a further cohort of mice at 69 weeks of age. No structural abnormalities, altered cardiac function or electrophysiological properties were detected using a combination of ECG under anesthesia, ambulatory ECG and *in vivo* electrophysiology (Table S7). Note that in this older cohort, the genotype-specific increase in heart rate was no longer apparent; rather, there was a trend to a decreased heart rate in mutant mice (Table S7). Taken together, these data indicate the lack of any major cardiac defect in mice up to 69 weeks of age, but evidence for an altered heart rate implicating an impact of the *HTT* mutation on the autonomic nervous system (ANS).

Lung function was assessed at 18 weeks of age using whole body plethysmography to determine spontaneous breathing patterns at activity and rest. During activity, male *HdhQ111/*+ mice exhibited an 8% increase in tidal volume (TV) compared to Hdh+/+ littermates (t-test male mutant versus wild-type p<0.05; Figure 8B), accompanied by secondary increases in inspiratory flow rates and minute volumes (the product of the TV and respiratory rate) (Table S8). Males also showed a trend towards increased TV at rest, but this did not reach statistical significance, likely because of the small number of mice that actually showed resting behavior (Table S8). Higher TVs may be associated with increased sympathetic nervous system activation [46], consistent with the increased heart rate in young mice; however increased tidal volume was only observed in males, and other signs of increased sympathetic activity such as an elevated respiratory rate were not apparent (Table S8). Together, these data provide evidence of mild cardiovascular and lung abnormalities present by 18-19 weeks of age and suggest a potential impact of the *HTT* CAG mutation on the autonomic nervous system. 

## Discussion

Here we present results of a phenotyping screen carried out in the accurate genetic *HdhQ111* knock-in mouse model of HD with the aim of uncovering phenotypes elicited by a single mutant *HTT* allele mimicking the genetic defect in the majority of HD patients. This is the first attempt at a comprehensive screen of an HD mouse model aimed at an extensive characterization across multiple physiological systems. It is important to note that in these standardized screens each phenotypic test is carried out at a specific age(s). Therefore, the screen does not reveal information on the progression of any particular mutant phenotype over time or on its potentially transient nature. Conversely, a negative result does not necessarily preclude a particular phenotype/physiological system from the pathogenic process in *HdhQ111* mice as the screen may not have captured an appropriate time-window for its detection. These factors notwithstanding, our phenotypic screen revealed a number of interesting phenotypes in heterozygous *HdhQ111* mice (Table 1); most of the deficits were behavioral, however cardiovascular, lung, and plasma metabolite abnormalities were also found. These findings affirm a growing body of data that the *HTT* CAG mutation results in an altered cellular state, and that while the brain/certain brain regions may be particularly susceptible to this altered state, deficits are also manifest in other tissues. Peripheral phenotypes are of considerable interest in HD as these can provide insight into disease mechanism in readily accessible tissues and have the potential to lead to biomarkers of disease onset/progression. 

### Behavioral abnormalities in HdhQ111/+ mice

While behavioral phenotypes have been examined in a number of knock-in HD mouse models to date [19-25,47-58] many of these previous studies have concentrated on phenotypes exhibited by homozygous mutant mice. Focusing on the phenotypes previously identified in heterozygous knock-in mice as the most relevant comparison to those in the present study, there appears to be good overall agreement between the models. In summary, heterozygous *HdhQ175* mice (C57BL/6J) exhibited decreased rearing, apparent at 16 and 32 weeks of age, hypoactivity from 20 weeks of age, a rotarod deficit, apparent at 30 and 38 weeks of age and general neurological abnormalities by 93 weeks of age [57]. Heterozygous *HdhQ140* mice (C57BL/6), from which the *HdhQ175* mice were derived, showed decreased rearing at 12 weeks of age and a rotarod deficit at 48 weeks of age [55]. Heterozygous *HdhQ200* mice (C57BL/6) exhibited a motor defect on the balance beam at 50 weeks of age, gait abnormalities at 60-80 weeks of age, altered grip strength at 80 weeks of age, by which time the mice were moribund [54]. A similar pattern of heterozygous phenotypes was seen in the *HdhQ150* line from which *HdhQ200* was derived, but at later ages [48,51]. Thus, in general, hypoactivity and motor coordination deficits appear relatively early, with gait abnormalities and more general neurological abnormalities apparent at later ages. Our observations in B6J.*HdhQ111*/+ mice are consistent with this general pattern. They also highlight learning/memory deficits, and uncover both novel behavioral phenotypes as well as some of the earliest behavioral abnormalities identified to date in heterozygous knock-in mice. 

The earliest behavioral abnormality we detected was increased time spent in the center of the open field arena, suggestive of decreased anxiety. Consistent with this was a trend towards decreased anxiety in the light/dark box. Both decreased [59,60] and increased [19,52,61] anxiety have previously been reported in various rodent HD models tested in a number of different paradigms. Our data indicate that decreased anxiety may be a very early response to a single mutant *HTT* allele, which, as previously hypothesized [59], may reflect the stimulation of repair mechanisms such as the release of anxiolytic neurotrophic factors or a hypercompensatory response. An early compensatory response is also indicated by a slight increase in motor learning on the rotarod in *HdhQ111* mice, reminiscent of a transient increase in motor performance or activity previously observed in other HD rodent models [49,59]. 

A deficit in the social discrimination task in male *HdhQ111* mice suggests that these mice have impaired social recognition memory. Memory deficits, including recognition memory deficits occur early in HD [32,62-64] and *HdhQ111*/+ mice have previously been shown to exhibit defects in long-term recognition memory and spatial memory by 4 months and 8 months of age respectively [56]. Note that we do not have evidence supporting a deficit in novel or familiar object recognition (data not shown), indicating that the recognition deficit uncovered in this screen is likely linked to the acquisition of olfactory memories. Social recognition memory in mice depends on two neuronal pathways for the acquisition and processing of olfactory cues: in one pathway sensory information from the olfactory epithelium is relayed through the main olfactory bulb (MOB) to higher brain areas that include the cortex and hippocampus. In the second pathway sensory information from the vomeronasal organ is relayed through the accessory olfactory bulb (AOB) to the amygdala, bed nucleus of stria terminalis and hypothalamus [65]. As *HdhQ111* mice also exhibited a deficit in their ability to distinguish two different odors it is possible that impairment in the social discrimination task reflects an olfaction deficit, rather than a memory deficit. However, given that both males and females exhibited an olfaction deficit with the phenotype appearing slightly more severe in females (Figure 6), this is perhaps unlikely to explain the male-specific defect in social discrimination. Furthermore, while sensory information from the odors to which the mice are exposed in the olfactory test are processed via the MOB pathway, short-term memory, as assayed in the social discrimination task described here, has a greater contribution from the AOB pathway [66]. This suggests that the inability to distinguish a novel and familiar mouse is likely to be due to a defect in recognition memory that may reflect an underlying deficit in the AOB pathway in *HdhQ111* mice. 

*HdhQ111* mice also exhibited markedly reduced odor discrimination without any loss of odor sensitivity. Olfaction deficits are well documented in HD as well as in several other neurodegenerative diseases, and in HD occurs in premanifest gene carriers 15-20 years prior to disease onset [33-38,67,68]. Interestingly, in some studies, *HTT* gene-positive individuals manifested either a specific defect or a more pronounced defect in odor discrimination relative to other olfactory measures [35,36], paralleling our findings in the mice. The absence of an alteration in smell sensitivity, together with a deficit during the training period, indicate that impaired olfactory learning, rather than impaired olfactory perception, might more likely underlie the odor discrimination phenotype in *HdhQ111* mice. Reduced olfactory memory in female mutants, together with a more pronounced training deficit in female mutants suggests that these phenotypes may result from common underlying deficit(s). 

The human caudate plays a role in odor discrimination [69], suggesting that pathogenesis occurring in HD caudate may contribute to this olfactory deficit. Of note, *HdhQ111* mice exhibit a time-dependent accumulation of mutant huntingtin in the nucleus in specific brain regions. The striatum is the first region in which this is observed, closely followed by the olfactory tubercle and piriform cortex [11] that receive direct input from the main olfactory bulb [65,70]. While the underlying cause and significance of nuclear huntingtin *per se* remains unclear, it reflects an ongoing pathogenic process [12] and its relation to olfactory deficit in *HdhQ111* mice would be of interest to understand. 

Interestingly, some odor discrimination tasks and olfactory learning are dependent on adult-born olfactory bulb interneurons [71-73 ] that arise from a proliferative stem cell population in the subventricular zone of the lateral ventricle [74]. Therefore, the impaired olfactory discrimination in *HdhQ111* mice may reflect abnormal olfactory bulb neurogenesis and/or a defect in the function of these adult-born neurons. Altered neurogenesis has been found in several HD mouse models including *HdhQ111* [36,61,75-80]. Odor discrimination may also be impaired by perturbations to olfactory sensory neurons [81]. Further experiments will be needed to understand the cellular and molecular underpinnings of the olfactory discrimination phenotype in *HdhQ111* mice. 

### Peripheral abnormalities in HdhQ111/+ mice

We identified a subtle but consistent decrease in free-fed blood glucose in female *HdhQ111*/+ mice. Our results demonstrate that in mice up to 19 weeks of age, a single mutant *HTT* allele does not induce diabetes or impaired glucose tolerance as seen in some patients as well as in N-terminal *HTT* fragment transgenic mice [42-44,82,83]. However, diabetes/glucose intolerance has not been seen in all HD patient cohorts examined, and it has been argued that these phenotypes may be more likely associated with more severe disease [84]. Our results indicate that rather than being glucose intolerant, young *HdhQ111*/+ mice exhibit signs of mildly increased glucose tolerance. 

Blood glucose levels are controlled by the opposing actions of insulin that stimulates glucose uptake, glycolysis, glycogen synthesis and inhibits gluconeogenesis and counterregulatory hormones, principally glucagon, adrenaline, growth hormone and glucocorticoids that promote gluconeogenesis and glycogenolysis, mainly in the liver. Interestingly, a similar, albeit more severe phenotype of reduced blood glucose in a fed but not in a fasting state was seen in mice carrying a liver-specific deletion of the gene encoding the stimulatory G protein α subunit, thereby inhibiting glucagon signaling in the liver [85]. These mice exhibited increased glucose tolerance and insulin sensitivity in the fed state, but were able to maintain normal glucose levels in the fasting state despite impaired glucagon signaling, likely due to excess glycogen stores, increased signaling by other counterregulatory hormones or sympathetic nervous system activity. These mice also exhibited a reduced fat content of which a trend was observed in *HdhQ111*/+ mice. Therefore, we speculate that in *HdhQ111*/+ mice a mild defect in gluoconegenesis induces a state of glucose tolerance in the fed state that can be compensated under fasting conditions where counterregulatory mechanisms dominate. Of interest, decreased gluconeogenesis has been proposed to account for the inability of HD patients to increase their blood glucose levels following exercise [86]. 

While the effects observed in young *HdhQ111*/+ mice are small, weight loss apparent by 46 weeks of age supports the occurrence of slowly-progressing underlying metabolic disturbances. Of note, the weight loss in *HdhQ111*/+ mice was more prominent in females, an effect also observed in two other lines of heterozygous knock-in mice (onset weight loss: *HdhQ175*/+ females 15 weeks, males 28 weeks [57]; *HdhQ200*/+ 50 weeks, males maintained their weight up to 80 weeks [54]). It is unclear at this point whether this is related to the greater tendency towards decreased glucose in female than male *HdhQ111*/+ mice (Figure 7). Taken together, however, our data hint that liver metabolism may be altered in *HdhQ111*/+ mice. That the *HTT* CAG mutation impacts the liver is indicated by late stage liver pathology in HD patients [87], the presence of nuclear inclusions in hepatocytes in HD mouse models [88,89] altered liver circadian function in vivo [90], and more recently, direct evidence for reduced liver function in both presymptomatic and symptomatic HD patients [91]. It is also worth noting that the liver, together with the striatum, exhibits the highest levels of somatic *HTT* CAG expansion in *HdhQ111* mice, predicted to accelerate CAG length-dependent phenotypes in this tissue [27]. Our results, together with previous observations indicating altered metabolism in HD knock-in mice [6,92-95], indicate that further investigation of the metabolic state of both the liver and other tissues would be of great interest. 

We observed a small effect of the *HdhQ111* mutation on bone mineral density, with a decrease in mutant females and an increase in mutant males. Decreased bone mineral density has been observed in premanifest *HTT* mutation carriers [96] and in the R6/2 HD transgenic mouse model [97] as well as in other neurodegenerative diseases [98]. With the exception of testosterone, the levels of which were unchanged in the mice in this study (data not shown), factors that influence bone mineral density, such as glucocortocoids, leptin, vitamin D were not measured in this screen. Further studies will be needed to understand the mechanisms underlying the putative bone remodeling in *HdhQ111* mice, including the possible role of the CNS [99]. 

Cardiovascular and lung phenotypes suggest altered ANS function, being most consistent with increased sympathetic activity at young ages. There is considerable evidence for ANS dysfunction in both manifest and premanifest HD individuals [41,100-105]. In HD individuals cardiovascular autonomic tests including blood pressure response to sustained hand-grip, orthostatic blood pressure test, heart rate and heart rate variability at rest and in response to maneuvers or stressors revealed deficits in both the sympathetic and parasympathetic branches of the ANS [41,100-105]. Our results support findings of altered cardiovascular parameters in other HD mouse models [106-108]; notably an increased heart rate was exhibited by both R6/1 and BACHD models [107,108]. Cardiac phenotypes are of interest as early-onset cardiac disease is the second leading cause of death in HD patients [109,110]. To our knowledge, there has been no previous report of altered lung function in HD. ANS deficits in HD are hypothesized to originate in the central autonomic network, consistent with cortical and hypothalamic pathology in the disease [41,104,111-113]. It is worth noting that blood glucose levels are also under the control of the ANS; thus an alternative hypothesis to explain the slightly depressed glucose levels seen in *HdhQ111* mice may be subtle alterations in centrally-mediated glucose sensing [114]. Our results prompt further studies to assess ANS dysfunction and underlying mechanisms in *HdhQ111* mice. 

## Conclusions

We have identified a number of phenotypes between 10 and ~50 weeks of age in *HdhQ111* mice, an accurate genetic model of HD. Significantly, phenotypes were elicited by a single full-length mutant *HTT* allele, as occurs in the majority of HD patients. It is of interest that many of the phenotypes observed were either sex-specific or more pronounced in one sex than another. Sexually dimorphic effects of mutant huntingtin have been previously observed in mice [61,115,116], and understanding the basis for this would likely provide insight into disease pathways. An ongoing goal of studies in *HdhQ111* mice is to identify reproducible early disease-relevant phenotypes for testing of genetic and pharmacological modifiers. Early phenotypes in accurate genetic knock-in mice are predicted to be subtle, as indicated by many of the phenotypes in the present study. While subtle phenotypes would not necessarily preclude their use as endpoints in modifier testing, further testing across multiple cohorts and at multiple ages to identify the most robust phenotypes would be of value. Importantly, assessment of these and other phenotypes in *Hdh* CAG allelic series in which mice differ in CAG repeat length will be important in prioritizing those phenotypes that are most relevant to the CAG length-dependent pathogenic process in patients. Future studies will also be aimed at a deeper understanding of the phenotypes uncovered in this screen at the cellular and molecular level, with the aim of revealing potential novel therapeutic targets. . 

## Supporting Information

Figure S1 **Acoustic startle response and prepulse inhibition in *HdhQ111*/+ and wild-type mice.** Acoustic startle response (ASR) (top graph) and prepulse inhibition (PPI) (bottom graph) were measured at 13 weeks of age. ASR was measured at background noise (NS) and sound pressure intensities of 70-120 dB. Note that there were small decreases in ASR response in male and female *HdhQ111*/+ mice, but these did not reach statistical significance. Sensorimotor gating was measured by PPI at a startle intensity of 110 dB and prepulse intensities of 67, 69, 73 and 81 dB. “Global” is the mean PPI value of all 4 prepulse intensities. N=20 *HdhQ111*/+ mice (10 males, 10 females) and N=21 Hdh+/+ mice (11 males, 10 females). Data points and bars represent mean±SEM.
(TIF)

Figure S2 **An olfactory deficit in *HdhQ111*/+ mice in an additional cohort of males.** Mice were trained on a scent [S+] (either strawberry or apple) and then tested for their ability to recognize decreasing proportions of this scent in a binary mixture. Test carried out at MGH on males at 24-27 weeks of age. N=10 per genotype.) Error bars show SEM. * p<0.05; ** p<0.01 in 2-tailed unpaired Student’s t-test comparing mutant and wild-type mice at each dilution.
(TIF)

Figure S3 **Test of olfactory memory.** Mice that had undergone the olfactory discrimination test (GMC pipeline 3) were re-tested for their ability to recognize the odorant on which they had been initially trained [S+] after an interval of either 16-17 weeks (A, C; mice trained to recognize strawberry) or 20-22 weeks (B, D; mice trained to recognize apple) following the end of the initial testing period. The bar graphs show the percentage of mice that correctly recognized [S+] after an interval of either 16-17 weeks of 20-22 weeks. Each graph represents data from 5 Hdh+/+ mice (white bars) and 5 *HdhQ111*/+ mice (grey bars). Note that there is no intrinsic difference between the apple and the strawberry odor in learning acquisition during initial training (data not shown). However, it is unknown whether there are odor-specific differences that relate to long-term olfactory memory, and therefore unclear whether apple-trained mice might also exhibit a memory deficit at 16-17 weeks of age. Note that these data are obtained from a single trial per mouse for 5 mice of each sex and genotype. Therefore, although all 5 tested Hdh+/+ females made the correct choice at 20 weeks, while all *HdhQ111*/+ females made the incorrect choice, this should not be interpreted as active avoidance of the “correct” odor in the *HdhQ111*/+ mice, but rather consistent with a possible difference in olfactory memory in the two genotypes that would need to be followed up with further experiments with additional mice and averaging several trials per mouse.
(TIF)

Figure S4 **Intraperitoneal glucose tolerance test.** An intraperitoneal glucose tolerance test (IpGTT) was performed at 14 weeks of age. Following fasting for 16 to 18 hours overnight mice were injected intraperitoneally with 2 g of glucose/kg body weight using a 20% glucose solution. Blood glucose was measured 15, 30, 60, 90 and 120 minutes after glucose injection. N=10 per group. Plotted are mean values ±SEM.
(TIF)

Table S1 **Tests performed in GMC pipelines of mice.**
(DOCX)

Table S2 **Behavioral tests in MGH mouse cohorts.**
(DOCX)

Table S3 **Open field testing in the dark phase in *HdhQ111*/+ and Hdh+/+ mice at 56-59 weeks of age.**
(DOCX)

Table S4 **Body weight measurements in *HdhQ111*/+ versus wild-type mice.**
(DOCX)

Table S5 **Clinical chemistry parameters in fed and fasted *HdhQ111*/+ versus wild-type mice.**
(DOCX)

Table S6 **Cardiovascular parameters in *HdhQ111*/+ versus wild-type mice at 12 and 19 weeks.**
(DOCX)

Table S7 **Cardiovascular parameters in *HdhQ111*/+ versus wild-type mice at 69 weeks.**
(DOCX)

Table S8 **Lung function parameters in *HdhQ111*/+ versus wild-type mice.**
(DOCX)

methods S1 (DOCX)
