# Supplementary figures and images for: A Broad Phenotypic Screen Identifies Novel Phenotypes Driven by a Single Mutant Allele in Huntington’s Disease CAG Knock-In Mice
Source: PLoS One. 2013 Nov 22;8(11):e80923. doi: 10.1371/journal.pone.0080923 (PMC3838378; doi:10.1371/journal.pone.0080923)

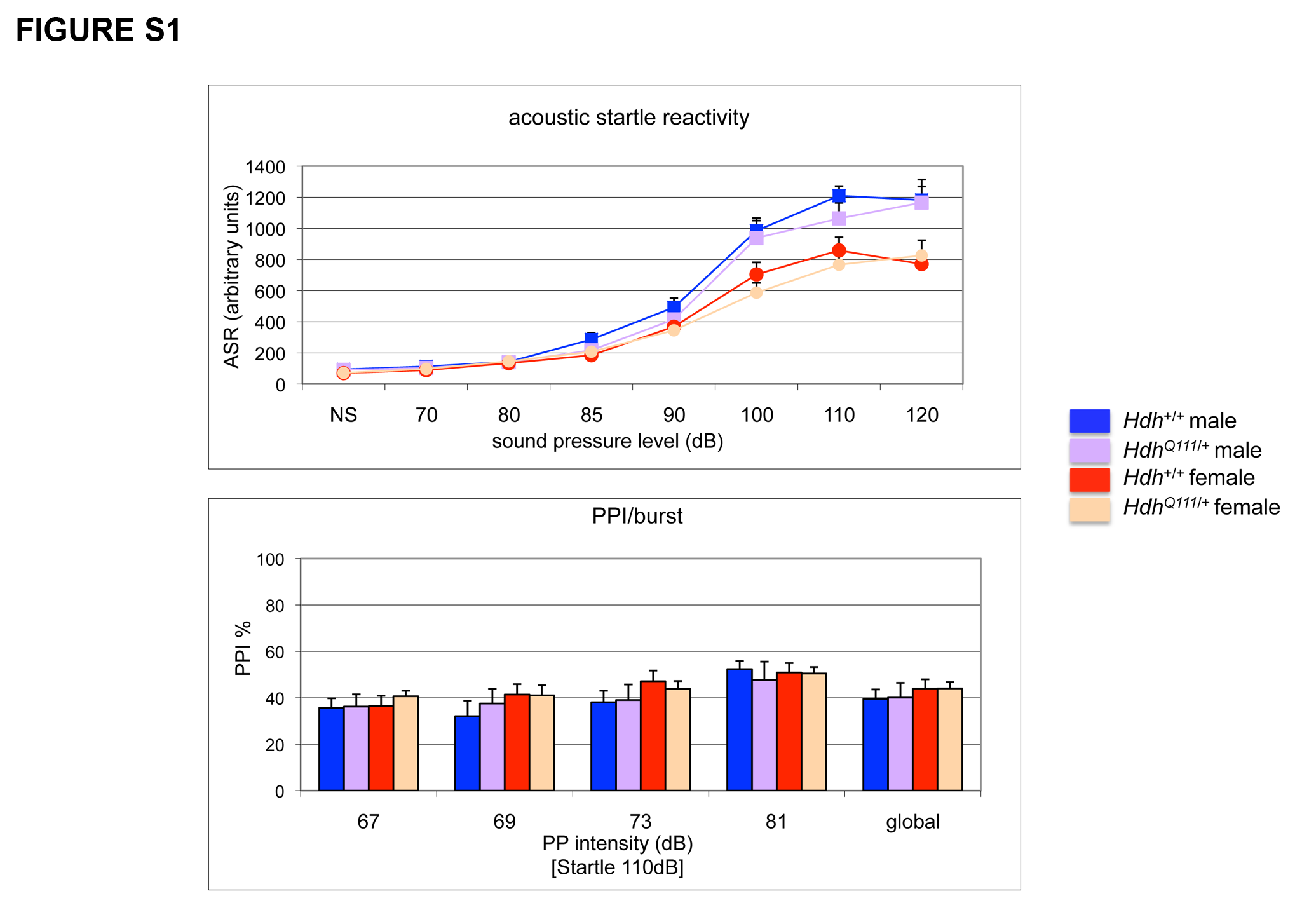

Supplement: Figure S1 — Acoustic startle response and prepulse inhibition in HdhQ111/+ and wild-type mice. Acoustic startle response (ASR) (top graph) and prepulse inhibition (PPI) (bottom graph) were measured at 13 weeks of age. ASR was measured at background noise (NS) and sound pressure intensities of 70-120 dB. Note that there were small decreases in ASR response in male and female HdhQ111/+ mice, but these did not reach statistical significance. Sensorimotor gating was measured by PPI at a startle intensity of 110 dB and prepulse intensities of 67, 69, 73 and 81 dB. “Global” is the mean PPI value of all 4 prepulse intensities. N=20 HdhQ111/+ mice (10 males, 10 females) and N=21 Hdh+/+ mice (11 males, 10 females). Data points and bars represent mean±SEM. (TIF) [file pone.0080923.s001.tif]

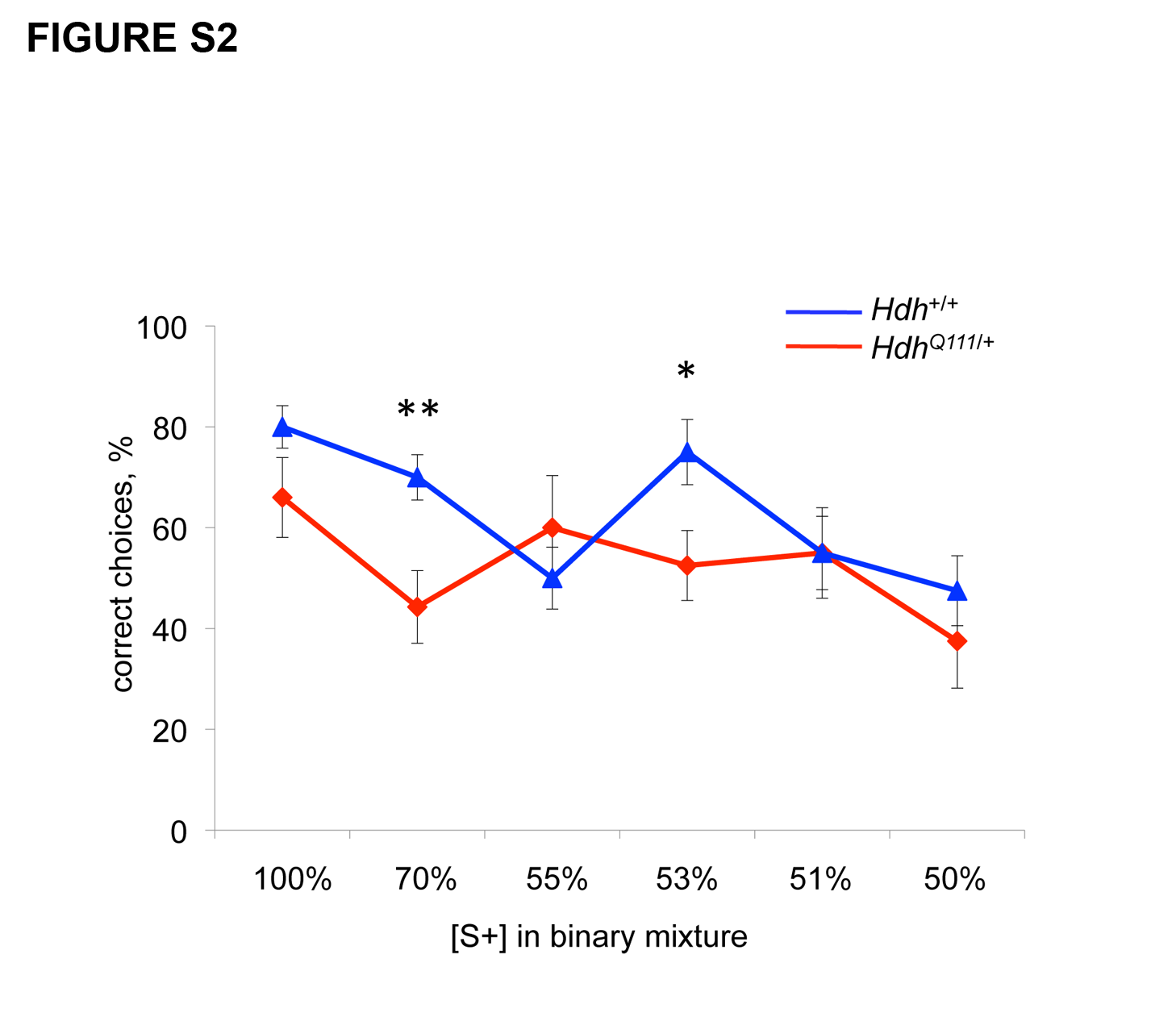

Supplement: Figure S2 — An olfactory deficit in HdhQ111/+ mice in an additional cohort of males. Mice were trained on a scent [S+] (either strawberry or apple) and then tested for their ability to recognize decreasing proportions of this scent in a binary mixture. Test carried out at MGH on males at 24-27 weeks of age. N=10 per genotype.) Error bars show SEM. * p<0.05; ** p<0.01 in 2-tailed unpaired Student’s t-test comparing mutant and wild-type mice at each dilution. (TIF) [file pone.0080923.s002.tif]

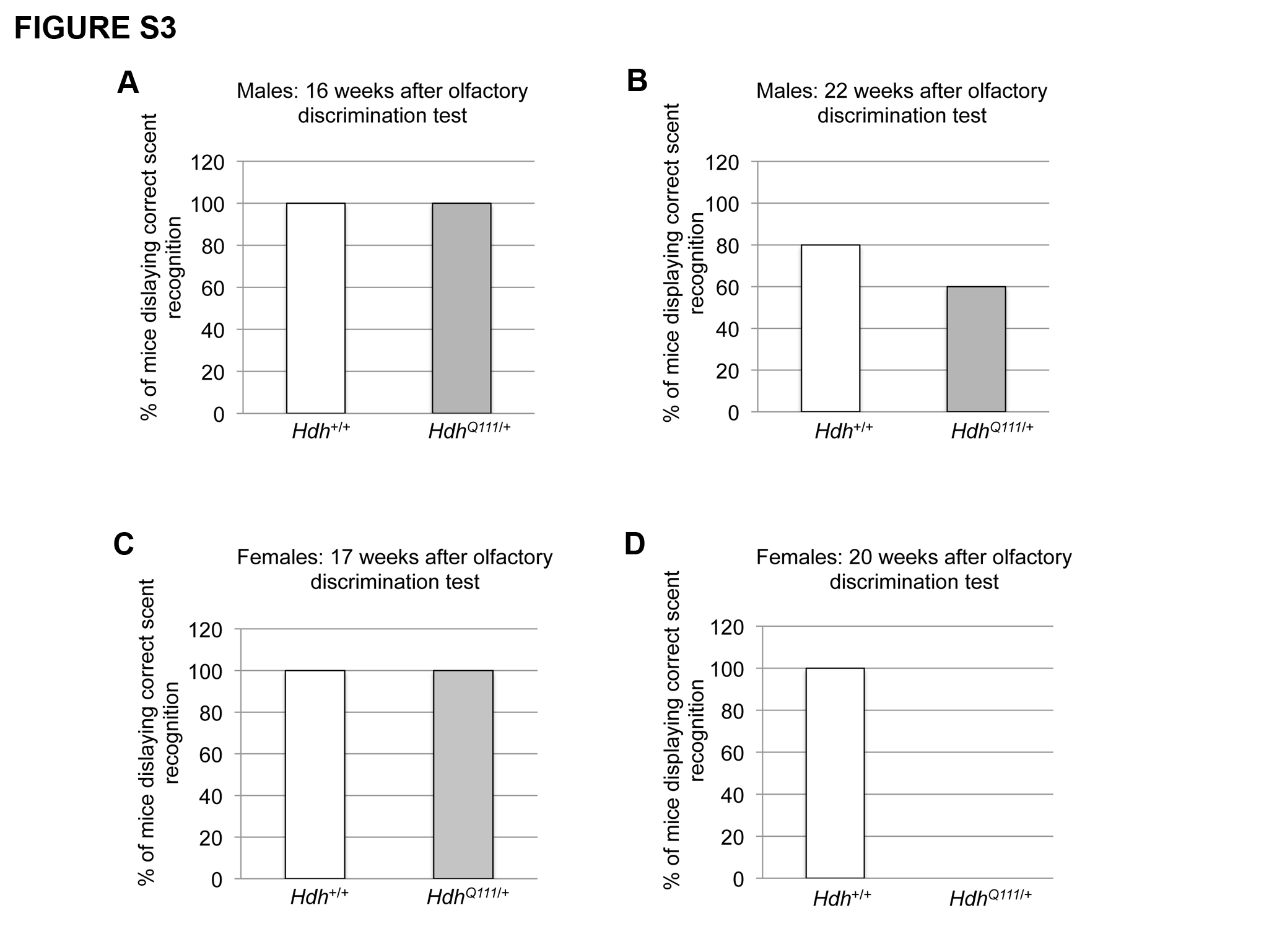

Supplement: Figure S3 — Test of olfactory memory. Mice that had undergone the olfactory discrimination test (GMC pipeline 3) were re-tested for their ability to recognize the odorant on which they had been initially trained [S+] after an interval of either 16-17 weeks (A, C; mice trained to recognize strawberry) or 20-22 weeks (B, D; mice trained to recognize apple) following the end of the initial testing period. The bar graphs show the percentage of mice that correctly recognized [S+] after an interval of either 16-17 weeks of 20-22 weeks. Each graph represents data from 5 Hdh+/+ mice (white bars) and 5 HdhQ111/+ mice (grey bars). Note that there is no intrinsic difference between the apple and the strawberry odor in learning acquisition during initial training (data not shown). However, it is unknown whether there are odor-specific differences that relate to long-term olfactory memory, and therefore unclear whether apple-trained mice might also exhibit a memory deficit at 16-17 weeks of age. Note that these data are obtained from a single trial per mouse for 5 mice of each sex and genotype. Therefore, although all 5 tested Hdh+/+ females made the correct choice at 20 weeks, while all HdhQ111/+ females made the incorrect choice, this should not be interpreted as active avoidance of the “correct” odor in the HdhQ111/+ mice, but rather consistent with a possible difference in olfactory memory in the two genotypes that would need to be followed up with further experiments with additional mice and averaging several trials per mouse. (TIF) [file pone.0080923.s003.tif]

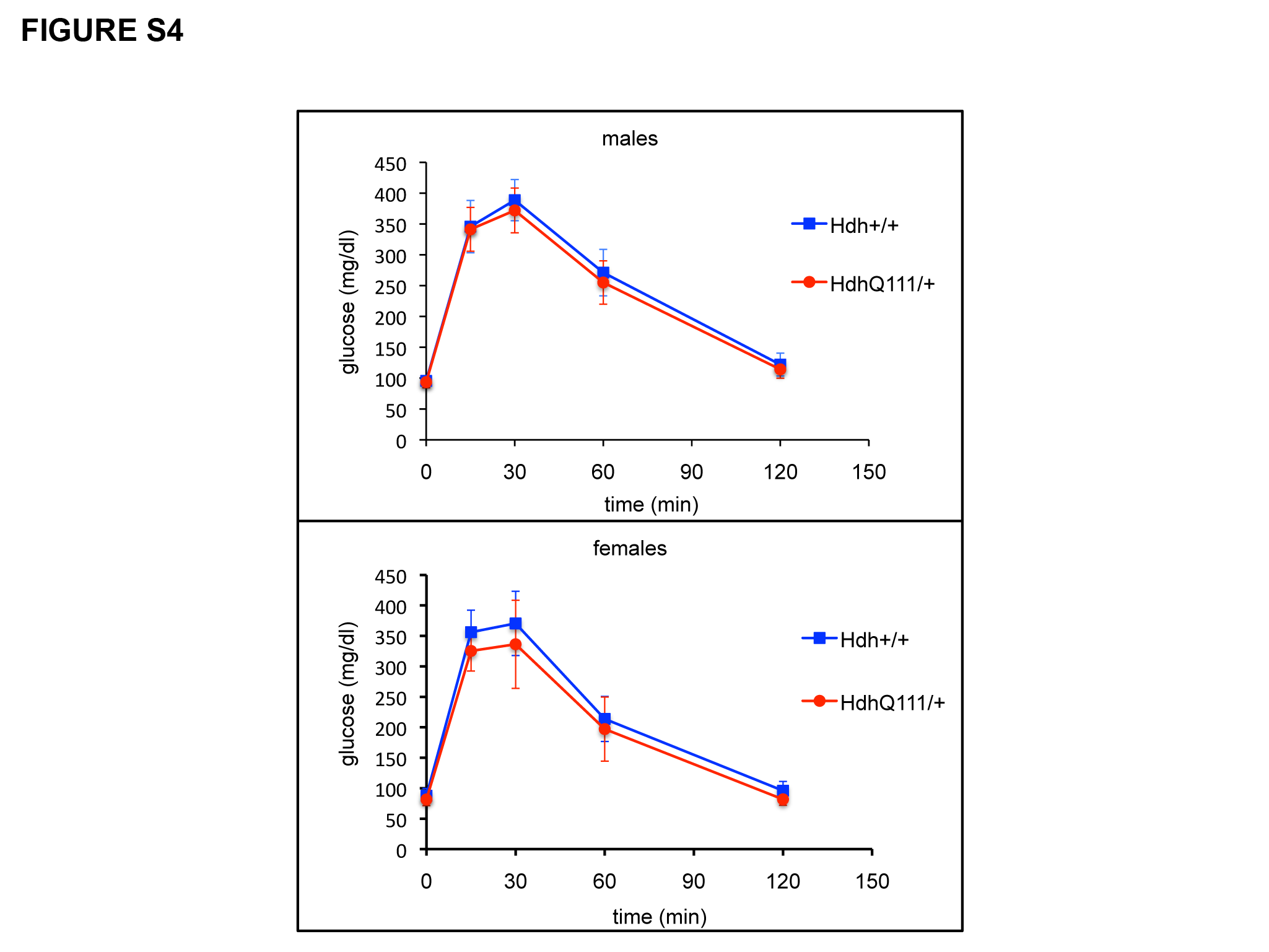

Supplement: Figure S4 — Intraperitoneal glucose tolerance test. An intraperitoneal glucose tolerance test (IpGTT) was performed at 14 weeks of age. Following fasting for 16 to 18 hours overnight mice were injected intraperitoneally with 2 g of glucose/kg body weight using a 20% glucose solution. Blood glucose was measured 15, 30, 60, 90 and 120 minutes after glucose injection. N=10 per group. Plotted are mean values ±SEM. (TIF) [file pone.0080923.s004.tif]
