# Supplementary material for: A Broad Phenotypic Screen Identifies Novel Phenotypes Driven by a Single Mutant Allele in Huntington’s Disease CAG Knock-In Mice
Source: PLoS One. 2013 Nov 22;8(11):e80923. doi: 10.1371/journal.pone.0080923 (PMC3838378; doi:10.1371/journal.pone.0080923)
Supplement: Table S1 — Tests performed in GMC pipelines of mice. (DOCX) [file pone.0080923.s005.docx]

**Table S1. Tests performed in GMC pipelines of mice**

| GMC pipeline 1 | |
| --- | --- |
| **Test** | **Age (weeks)** |
| visual morphological observation, hearing | 10 |
| blood pressure | 12 |
| calorimetry | 13 |
| simplified IpGTT | 14 |
| X-ray and DEXA | 15-16 |
| fasted mice blood plasma values | 16 |
| eye size (laser interference biometry) | 17 |
| spontaneous breathing pattern | 18 |
| GMC pipeline 2 | |
| **Test** | **Age (weeks)** |
| grip strength and modified SHIRPA | 11 |
| open field | 11 |
| Rotarod, vertical pole | 12 |
| acoustic startle and pre-pulse inhibition | 13 |
| ophthalmoscope and slit lamp | 15 |
| clinical chemistry and hematology | 16 |
| ANP, ECG, heart weight | 19 |
| clinical chemistry and hematology | 19 |
| macroscopic and histological analyses | 19 |
| GMC pipeline 3 | |
| **Test** | **Age (weeks)** |
| light/dark box | 11 |
| social discrimination | 14-16 |
| olfaction | 28-36 |
| gait | 46 |
| vertical pole | 46 |

Phenotyping performed at the GMC (see [www.mouseclinic.de](http://www.mouseclinic.de).). IpGTT: intraperitoneal glucose tolerance test: DEXA: dual-energy X-ray absorptiometry; FACS: fluorescence-activated cell sorting; ANP: plasma N-terminal atrial natriurietic peptide.
