# Supplementary material for: A Broad Phenotypic Screen Identifies Novel Phenotypes Driven by a Single Mutant Allele in Huntington’s Disease CAG Knock-In Mice
Source: PLoS One. 2013 Nov 22;8(11):e80923. doi: 10.1371/journal.pone.0080923 (PMC3838378; doi:10.1371/journal.pone.0080923)
Supplement: Table S2 — Behavioral tests in MGH mouse cohorts. (DOCX) [file pone.0080923.s006.docx]

**Table S2. Behavioral tests in MGH mouse cohorts**

| MGH cohort 1 | |
| --- | --- |
| **tests** | **Age (weeks)** |
| Open field (light) | 10 |
| Olfactory discrimination (males) | 24-27 |
| Open field (dark) | 40 |
| MGH cohort 2 | |
| **test** | **Age (weeks)** |
| Open field (light) | 9 |
| Rotarod | 10 |
| Rotarod | 24 |
| MGH cohort 3 | |
| **test** | **Age (weeks)** |
| Open field (light) | 11 |
| Olfactory discrimination | 42-47 (males)  50-56 (females) |
| Open field (dark) | 56-59 |
