# Supplementary material for: A Broad Phenotypic Screen Identifies Novel Phenotypes Driven by a Single Mutant Allele in Huntington’s Disease CAG Knock-In Mice
Source: PLoS One. 2013 Nov 22;8(11):e80923. doi: 10.1371/journal.pone.0080923 (PMC3838378; doi:10.1371/journal.pone.0080923)
Supplement: Table S3 — Open field testing in the dark phase in HdhQ111/+ and Hdh+/+ mice at 56-59 weeks of age. (DOCX) [file pone.0080923.s007.docx]

**Table S3. Open field testing in the dark phase in *HdhQ111*/+ and *Hdh*+/+ mice at 56-59 weeks of age**

| **parameter** | **males** | | **females** | | **p value** |
| --- | --- | --- | --- | --- | --- |
|  | ***Hdh*+/+** | ***HdhQ111*/+** | ***Hdh*+/+** | ***HdhQ111*/+** |  |
| Time in center (s) | 140±20 | 178±33 | 125±22 | 121±13 | 0.53 |
| Distance traveled in center (cm) | 642±62 | 463±68 | 911±160 | 548±39 | 0.004 |
| Distance traveled in periphery (cm) | 1800±166 | 1445±113 | 3076±316 | 2144±160 | 0.008 |
| Velocity in center (cm/s) | 5.04±0.46 | 3.03±0.51 | 7.46±0.68 | 4.96±0.48 | 0.0005 |
| Velocity in periphery (cm/s) | 1.69±0.15 | 1.43±0.12 | 2.88±0.31 | 1.99±0.15 | 0.012 |
| Ambulatory time in center (s) | 21.9±1.78 | 17.1±2.41 | 32.9±5.33 | 20.5±1.23 | 0.006 |
| Ambulatory time in periphery (s) | 64.7±6.14 | 50.5±4.47 | 116.0±12.9 | 81.8±6.8 | 0.015 |
| Stereotypy time in center (s) | 37.2±5.11 | 41.0±6.61 | 32.0±5.62 | 26.4±2.92 | 0.74 |
| Stereotypy time in periphery (s) | 255±10.7 | 231±11.9 | 277±10.4 | 247±4.57 | 0.018 |
| Vertical time in center (s) | 2.63±0.84 | 4.17±1.31 | 1.64±0.84 | 0.80±0.32 | 0.84 |
| Vertical time in periphery (s) | 83.8±9.20 | 82.7±10.3 | 83.9±10.07 | 68.2±4.20 | 0.31 |

Values given are mean±SEM. The effect of genotype on the open field parameters was assessed by 2-way ANOVA. p values shown are for genotype effect. Mice tested were MGH cohort 3 (see Table S2 and Materials and Methods).
