# Supplementary material for: A Broad Phenotypic Screen Identifies Novel Phenotypes Driven by a Single Mutant Allele in Huntington’s Disease CAG Knock-In Mice
Source: PLoS One. 2013 Nov 22;8(11):e80923. doi: 10.1371/journal.pone.0080923 (PMC3838378; doi:10.1371/journal.pone.0080923)
Supplement: Table S4 — Body weight measurements in HdhQ111/+ versus wild-type mice. (DOCX) [file pone.0080923.s008.docx]

**Table S4. Body weight measurements in *HdhQ111*/+ versus wild-type mice**

| **Age (wks)** | **males** | | **females** | |
| --- | --- | --- | --- | --- |
|  | ***Hdh*+/+** | ***HdhQ111*/+** | ***Hdh*+/+** | ***HdhQ111*/+** |
| 11 | 25.1±0.4 | 24.4±0.3 | 18.9±0.4 | 19.1±0.5 |
| 13 | 25.9±0.5 | 25.0±0.6 | 19.5±0.3 | 19.3±0.5 |
| 16 | 26.9±0.3 | 26.1±0.4 | 20.4±0.4 | 20.1±0.2 |
| 18 | 26.7±0.6 | 26.6±0.7 | 20.4±0.5 | 20.4±0.6 |
| 19 | 26.3±0.5 | 25.7±0.3 | 19.9±0.4 | 19.9±0.6 |
| *46 | 37.5±5.6 | 31.9±3.3 | 32.5±3.8 | 28.5±0.8 |

Body weight was measured in GMC pipeline 1 mice (no shading) at 13 weeks (metabolism screen, N=7 per group), 16 weeks (dysmorphology screen, N=10 per group) and 18 weeks (lung function screen, N=6 per group). Body weight was measured in GMC pipeline 2 mice (light grey shading) at 11 weeks (neurology screen, N=11 *Hdh*+/+ males and N=10 for all other groups) and at 19 weeks (cardiovascular screen, N=11 Hdh+/+ males, N=10 *HdhQ111*/+ males, N=9 *Hdh*+/+ females, N=7 *HdhQ111*/+ females). Body weight was measured in GMC pipeline 3 mice (dark grey shading) at 46 weeks (N=5 per group). * 2 tailed unpaired Student’s t-tests at 46 weeks: Hdh*Q111/+ versus Hdh+/+:* males p=0.09; females p=0.049. Values are mean±SEM.
