# Supplementary material for: A Broad Phenotypic Screen Identifies Novel Phenotypes Driven by a Single Mutant Allele in Huntington’s Disease CAG Knock-In Mice
Source: PLoS One. 2013 Nov 22;8(11):e80923. doi: 10.1371/journal.pone.0080923 (PMC3838378; doi:10.1371/journal.pone.0080923)
Supplement: Table S5 — Clinical chemistry parameters in fed and fasted HdhQ111/+ versus wild-type mice. (DOCX) [file pone.0080923.s009.docx]

**Table S5. Clinical chemistry parameters in fed and fasted *HdhQ111*/+ versus wild-type mice**

| **parameter** | **males** | | **females** | |
| --- | --- | --- | --- | --- |
|  | ***Hdh*+/+** | ***HdhQ111*/+** | ***Hdh*+/+** | ***HdhQ111*/+** |
| Clinical chemistry parameters in free-fed mice (1st sample) | | | | |
| Sodium (mM) | 148.7±0.67 | 148±0.82 | 142.5±1.05 | 145.1±1.14 |
| Potassium (mM) | 4.13 ± 0.09 | 4.16 ± 0.06 | 3.63 ± 0.14 | 3.97 ± 0.26 |
| Calcium (mM) | 2.16 ± 0.01 | 2.15 ± 0.01 | 2.09 ± 0.02 | 2.11 ± 0.02 |
| Chloride (mM) | 112 ± 0.63 | 111.8 ± 0.51 | 110.3 ± 0.79 | 111.8 ± 1.27 |
| Inorganic phosphate (mM) | 1.56 ± 0.05 | 1.5 ± 0.07 | 1.46 ± 0.08 | 1.62 ± 0.06 |
| Total protein (g/dl) | 4.94 ± 0.04 | 4.82 ± 0.05 | 4.58 ± 0.06 | 4.58 ± 0.06 |
| Albumin (g/dl) | 2.8 ± 0 | 2.74 ± 0.031 | 2.68 ± 0.033 | 2.64 ± 0.04 |
| Creatinine (mg/dl) | 0.1 ± 0.01 | 0.1 ± 0.01 | 0.1 ± 0.01 | 0.1 ± 0.01 |
| Urea (mg/dl) | 79.3 ± 1.96 | 72.5 ± 1.32 | 79.2 ± 2.67 | 76.9 ± 3.27 |
| #Cholesterol (mg/dl) | 97.2 ± 3.69 | 91 ± 4.37 | 72.5 ± 1.38 | 66.3 ± 3.19 |
| Triglycerides (mg/dl) | 158 ± 7.9 | 166 ± 14.7 | 104 ± 7.9 | 99 ± 7.3 |
| NEFA (mM) | 1.6 ± 0.16 | 1.6 ± 0.11 | 1.1 ± 0.05 | 0.9 ± 0.1 |
| LDH (U/l) | 241.1 ± 32.33 | 233.2 ± 33.56 | 234.5 ± 22.82 | 408.3 ±142.94 |
| ALAT (U/l) | 26.4 ± 2.25 | 29.2 ± 4.07 | 35.2 ± 3.89 | 31.6 ± 3.25 |
| ASAT (U/l) | 54 ± 8.92 | 55.8 ± 8.6 | 44.2 ± 2.36 | 53.6 ± 4.48 |
| AP (U/l) | 106.8 ± 2.13 | 100.4 ± 3.14 | 137.2 ± 3.35 | 137.8 ± 6.75 |
| α-amylase (U/l) | 681 ± 20 | 673 ± 27.5 | 562 ± 15.9 | 582 ± 12.9 |
| *Glucose (mg/dl) | 196 ± 12.3 | 197.8 ± 18 | 245.2 ± 8.9 | 211.3 ± 11.3 |
| Ferritin (ng/ml) | 24 ± 1.48 | 25.5 ± 0.9 | 29 ± 1.66 | 27.2 ± 1.57 |
| Transferrin (mg/dl) | 137.7 ± 1.07 | 134.9 ± 0.45 | 134.5 ± 1.57 | 134.7 ± 2.19 |
| Iron (µg/dl) | 116.5 ± 3.61 | 107.7 ± 3.33 | 114.9 ± 3.52 | 122.7 ± 8.84 |
| Serum lactate (mM) | 8.93 ± 0.42 | 10.07 ± 0.79 | 9.12 ± 0.29 | 9.86 0.45 |
| Clinical chemistry parameters in free-fed mice (2nd sample) | | | | |
| *Glucose (mg/dl) | 227.8 ± 14 | 202.2 ± 8.9 | 247 ± 8.8 | 215.1 ± 3.7 |
| Ferritin (ng/ml) | 14.4 ± 0.78 | 16.2 ± 1.07 | 23 ± 8.33 | 17.8 ± 1.25 |
| Transferrin (mg/dl) | 100.4 ± 1.15 | 99.6 ± 1.12 | 102.9 ± 1.66 | 99.7 ± 2.84 |
| Iron (µg/dl) | 114.5 ± 4.45 | 116.6 ± 4.44 | 105.9 ± 8.06 | 125.1 ± 10.13 |
| Clinical chemistry parameters in fasted mice | | | | |
| Cholesterol (mg/dl) | 98.8 ± 1.97 | 99.8 ± 3.47 | 77.1 ± 1.73 | 75.4 ± 1.6 |
| Triglycerides (mg/dl) | 91 ± 9 | 103 ± 12.7 | 98 ± 6.8 | 104 ± 8.1 |
| NEFA (mM) | 1.3 ± 0.05 | 1.5 ± 0.07 | 1.5 ± 0.07 | 1.5 ± 0.07 |
| Glucose (mg/dl) | 191.6 ± 11.2 | 193.7 ± 12.1 | 192.1 ± 13 | 206.7 ± 11.7 |

Measurements in free-fed mice (pipeline 2) were at 16 weeks (1st sample; n=10 for each sex and genotype) and again at 19 weeks for a subset of parameters (2nd sample; n=10 males for each genotype, n=9 *Hdh*+/+ females and n=7 *HdhQ111*/+ females). Measurements in fasted mice (pipeline 1) were at 16 weeks (n=10 for each sex and genotype). # Cholesterol levels in free-fed mice trended towards a decrease in mutants (ANOVA genotype effect p=0.071). * Glucose levels in free-fed mice was significantly decreased in females in both test samples (2-tailed unpaired Student’s t-test p<0.05). NEFA: non-esterified fatty acids; LDH: lactate dehydrogenase; ALAT: alanine aminotransferase; ASAT: aspartate aminotransferase; AP: alkaline phosphatase. Values are mean±SEM.
