# Supplementary material for: A Broad Phenotypic Screen Identifies Novel Phenotypes Driven by a Single Mutant Allele in Huntington’s Disease CAG Knock-In Mice
Source: PLoS One. 2013 Nov 22;8(11):e80923. doi: 10.1371/journal.pone.0080923 (PMC3838378; doi:10.1371/journal.pone.0080923)
Supplement: Table S6 — Cardiovascular parameters in HdhQ111/+ versus wild-type mice at 12 and 19 weeks. (DOCX) [file pone.0080923.s010.docx]

**Table S6. Cardiovascular parameters in *HdhQ111*/+ versus wild-type mice at 12 and 19 weeks**

| **parameter** | **males** | | **females** | |
| --- | --- | --- | --- | --- |
|  | ***Hdh*+/+** | ***HdhQ111*/+** | ***Hdh*+/+** | ***HdhQ111*/+** |
| Systolic pressure (mm Hg) | 115.4±2.3 | 119.1+/-1.9 | 127.0+/-3.0 | 123.9+/-3.3 |
| Diastolic pressure (mm Hg) | 101.9+/-2.5 | 106.5+/-2.0 | 114.8+/-2.5 | 110.6+/-3.3 |
| Mean arterial pressure (mm Hg) | 106.1+/-2.4 | 110.3+/-1.9 | 118.5+/-2.6 | 114.7+/-3.3 |
| Pulse (bpm) | 527.7+/-11.4 | 550.5+/-24.5 | 555.5+/-11.0 | 605.5+/-19.0 |
| Nt proANP (nM) | 0.71+/-0.07 | 0.75+/-0.08 | 1.61+/-0.20 | 1.71+/-0.24 |
| Body weight (g) | 26.3+/-0.5 | 25.7+/-0.3 | 19.9+/-0.4 | 19.9+/-0.6 |
| Heart weight (g) | 0.15+/-0.00 | 0.14+/-0.00 | 0.11+/-0.00 | 0.12+/-0.01 |
| Tibia length (mm) | 20.1+/-0.3 | 20.3+/-0.3 | 19.7+/-0.2 | 19.6+/-0.2 |
| Heart weight/tibia length | 7.43+/-0.21 | 6.85+/-0.20 | 5.67+/-0.10 | 6.11+/-0.47 |
| Heart weight/body weight | 5.66+/-0.12 | 5.66+/-0.13 | 5.66+/-0.10 | 6.05+/-0.54 |

Blood pressure and pulse measurements were at 12 weeks (n= 10 per group). Nt proANP was measured at 19 weeks (n= 10 per group apart from n=9 *HdhQ111*/+ females). Other measurements were at 19 weeks (n=11 *Hdh*+/+ males, n=10 *HdhQ111*/+ males, n=10 *Hdh*+/+ females, n=7 *HdhQ111*/+ females. Values are mean±SEM.
