# Supplementary material for: A Broad Phenotypic Screen Identifies Novel Phenotypes Driven by a Single Mutant Allele in Huntington’s Disease CAG Knock-In Mice
Source: PLoS One. 2013 Nov 22;8(11):e80923. doi: 10.1371/journal.pone.0080923 (PMC3838378; doi:10.1371/journal.pone.0080923)
Supplement: Table S7 — Cardiovascular parameters in HdhQ111/+ versus wild-type mice at 69 weeks. (DOCX) [file pone.0080923.s011.docx]

**Table S7. Cardiovascular parameters in *HdhQ111*/+ versus wild-type mice at 69 weeks**

|  | ***Hdh*+/+** | ***HdhQ111*/+** | **p value** |
| --- | --- | --- | --- |
| **Echocardiogram** |  |  |  |
| IVS (mm) | 0.79 ± 0.08 | 0.74 ± 0.04 | 0.35 |
| LVDd (mm) | 3.89 ± 0.26 | 3.91 ± 0.28 | 0.93 |
| LVPW (mm) | 0.79 ± 0.03 | 0.79 ± 0.03 | 0.85 |
| FS (%) | 29.6 ± 7.2 | 29.88 ± 3.9 | 0.95 |
| **Ambulatory ECG** |  |  |  |
| Pulse (bpm) | 667.4 ± 55.5 | 555.2 ± 104.4 | 0.09 |
| PR interval (msec) | 36.1 ± 2.4 | 38.4 ± 5.3 | 0.41 |
| QRS interval (msec) | 10.3 ± 1.7 | 11.2 ± 0.7 | 0.43 |

5 *Hdh*+/+ and 3 *HdhQ111*/+ mice were assessed for heart function by echocardiogram and by ambulatory ECG without anesthesia. *In vivo* electrophysiology was performed in a subset of animals (n=2 in each group), confirming the lack of significant differences between genotypes on either cardiac conduction properties or arrhythmogenicity.
