# Supplementary material for: A Broad Phenotypic Screen Identifies Novel Phenotypes Driven by a Single Mutant Allele in Huntington’s Disease CAG Knock-In Mice
Source: PLoS One. 2013 Nov 22;8(11):e80923. doi: 10.1371/journal.pone.0080923 (PMC3838378; doi:10.1371/journal.pone.0080923)
Supplement: Table S8 — Lung function parameters in HdhQ111/+ versus wild-type mice. (DOCX) [file pone.0080923.s012.docx]

**Table S8. Lung function parameters in *HdhQ111*/+ versus wild-type mice**

| **parameter** | **males** | | **females** | |
| --- | --- | --- | --- | --- |
|  | ***Hdh*+/+** | ***HdhQ111*/+** | ***Hdh*+/+** | ***HdhQ111*/+** |
| Baseline parameters n=6 n=6 n=6 n=6 | | | | |
| Bw [g] | 26.7 ± 0.6 | 20.4 ± 0.5 | 20.4 ± 0.5 | 20.4 ± 0.6 |
| Age [d] | 132.5 ± 0.2 | 131.5 ± 0.2 | 131.5 ± 0.2 | 131.7 ± 0.2 |
| Mean_f [1/min] | 444.1 ± 15.1 | 456.9 ± 12.1 | 456.9 ± 12.1 | 472.0 ± 11.3 |
| Respiratory rate and timing at rest and activity | | | | |
| Rest | n=4 | n=4 | n=4 | n=3 |
| f [1/min] | 323.6 ± 14.8 | 298.5 ± 15.3 | 329.1 ± 2.9 | 324.1 ± 2.7 |
| Ti [ms] | 55.6 ± 2.0 | 57.7 ± 3.5 | 49.7 ± 1.0 | 51.2 ± 0.3 |
| Te [ms] | 131.1 ± 7.5 | 144.8 ± 7.6 | 132.7 ± 0.9 | 133.9 ± 1.5 |
| Ti/TT | 0.30 ± 0.01 | 0.29 ± 0.01 | 0.27 ± 0.00 | 0.28 ± 0.00 |
| Activity | n=6 | n=6 | n=6 | n=6 |
| f [1/min] | 486.4 ± 5.4 | 494.6 ± 5.3 | 494.6 ± 5.3 | 500.7 ± 4.3 |
| Ti [ms] | 42.3 ± 0.6 | 41.9 ± 0.5 | 41.9 ± 0.5 | 42.0 ± 0.2 |
| Te [ms] | 81.1 ± 1.0 | 79.5 ± 1.1 | 79.5 ± 1.1 | 77.9 ± 1.1 |
| Ti/TT | 0.34 ± 0.00 | 0.34 ± 0.00 | 0.34 ± 0.00 | 0.35 ± 0.00 |
| Tidal volume and flow rates at rest and activity | | | | |
| Rest | n=4 | n=4 | n=4 | n=3 |
| TV [ml] | 0.21 ± 0.01 | 0.24 ± 0.01 | 0.21 ± 0.01 | 0.22 ± 0.00 |
| PIF [ml/s] | 6.6 ± 0.1 | 7.4 ± 0.5 | 7.2 ± 0.4 | 7.3 ± 0.1 |
| PEF [ml/s] | 3.4 ± 0.1 | 3.6 ± 0.3 | 3.5 ± 0.2 | 3.5 ± 0.1 |
| MIF [ml/s] | 3.8 ± 0.1 | 4.2 ± 0.3 | 4.3 ± 0.2 | 4.3 ± 0.1 |
| MEF [ml/s] | 1.6 ± 0.1 | 1.7 ± 0.1 | 1.6 ± 0.1 | 1.6 ± 0.0 |
| Activity | n=6 | n=6 | n=6 | n=6 |
| *TV [ml] | 0.24 ± 0.01 | 0.26 ± 0.01 | 0.22 ± 0.01 | 0.22 ± 0.00 |
| *PIF [ml/s] | 9.1 ± 0.3 | 10.2 ± 0.2 | 8.5 ± 0.4 | 8.7 ± 0.1 |
| PEF [ml/s] | 5.9 ± 0.3 | 6.5 ± 0.2 | 5.5 ± 0.2 | 5.6 ± 0.1 |
| *MIF [ml/s] | 5.6 ± 0.2 | 6.2 ± 0.1 | 5.3 ± 0.2 | 5.3 ± 0.1 |
| MEF [ml/s] | 2.9 ± 0.1 | 3.3 ± 0.1 | 2.8 ± 0.1 | 2.9 ± 0.0 |
| Minute ventilation and bodyweight related parameters at rest and activity | | | | |
| Rest | n=4 | n=4 | n=4 | n=3 |
| sTV [µl/g] | 7.9 ± 0.4 | 9.2 ± 0.7 | 10.6 ± 0.4 | 10.9 ± 0.4 |
| MV [ml/min] | 65.5 ± 2.8 | 70.3 ± 5.8 | 67.2 ± 3.4 | 69.3 ± 2.0 |
| sMV [ml/min/g] | 2.5 ± 0.1 | 2.7 ± 0.3 | 3.4 ± 0.1 | 3.5 ± 0.1 |
| Activity | n=6 | n=6 | n=6 | n=6 |
| sTV [µl/g] | 8.8 ± 0.3 | 9.8 ± 0.4 | 10.8 ± 0.4 | 11.0 ± 0.3 |
| *MV [ml/min] | 113.5 ± 4.4 | 127.6 ± 4.0 | 108.2 ± 4.9 | 111.5 ± 0.8 |
| sMV [ml/min/g] | 4.3 ± 0.2 | 4.8 ± 0.2 | 5.3 ± 0.2 | 5.5 ± 0.1 |

Lung function parameters were determined at 18 weeks of age. Bw: body weight: mean_f: mean of all respiratory rates; f: respiratory rate; TV: tidal volume; sTV: specific tidal volume; MV: minute ventilation; sMV: specific ventilation; Ti: inspiratory time; Te: expiratory time; Ti/TT: relative duration of inspiration; PIF: peak inspiratory flow rate; PEF: peak expiratory flow rate; MIF: mean inspiratory flow rate; MEF: mean expiratory flow rate. * 2-tailed unpaired Student’s t-tests showed significant genotype differences in males in TV (p<0.05), PIF (p<0.02), MIF (p<0.05) and MV (p<0.05) during activity. Similar trends were seen at rest but did not reach statistical significance, potentially due to the lower number of mice at rest. Refer to table for numbers of mice for each test. Values are mean±SEM.
