# Supplementary material for: A Broad Phenotypic Screen Identifies Novel Phenotypes Driven by a Single Mutant Allele in Huntington’s Disease CAG Knock-In Mice
Source: PLoS One. 2013 Nov 22;8(11):e80923. doi: 10.1371/journal.pone.0080923 (PMC3838378; doi:10.1371/journal.pone.0080923)
Supplement: methods S1 — (DOCX) [file pone.0080923.s013.docx]

**Analyses of morphology, ear and eye function**

Animals were screened using a protocol for morphological analysis from [[1](#_ENREF_1)]. Detailed protocols for screening for bone and cartilage phenotypes in mice are described in [[2](#_ENREF_2)]. Hearing was assessed using a clickbox, measuring reaction time to a sound of 20 kHz. X-ray analysis of anesthetized mice was performed using a Faxitron X-ray Model MX-20 (Specimen Radiography System, Illinois, USA) and NTB Digital X-ray Scanner EZ 40 (NTB GmbH, Diepholz, Germany) with settings of 25 kV and integration time 40 ms. Images were acquired using iX-Pect software. Bone density, lean and fat mass were measured in anesthetized mice using a DEXA Sabre X-ray Bone Densitometer (Norland Medical Systems. Inc., Basingstoke, UK, using a scan speed of 20 mm/s, and resolution of 0.5 mm x 1.0 mm, HAW 0.020. The posterior parts of both eyes were examined by funduscopy after pupil dilation using a head-worn indirect ophthalmoscope (Sigma 150 K, Heine Optotechnik, Herrsching, Germany) in conjunction with a condensing lens (90D lens, Volk, Mentor, OH, USA) mounted between the ophthalmoscope and the eye. Both eyes of the mice were examined by slit lamp biomicroscopy (Zeiss SLM30) at 48x magnification with a narrow beam slit lamp illumination at 25-30°angle from the direction of observation. Laser Interference Biometry (LIB) was performed on anesthetized mice using the AC Master (Meditec, Carl Zeiss, Jena, Germany) equipped with optical low coherence interferometry (OLCI), adapted for short measurement distances.

**Pathological assessment**

Mice were sacrificed with CO_2_ and organs were fixed in 4% buffered formalin and embedded in paraffin for histological examination. Two-μm-thick sections from skin, heart, muscle, lung, brain, cerebellum, thymus, spleen, cervical lymph nodes, thyroid, parathyroid, adrenal gland, stomach, intestine, liver, pancreas, kidney, reproductive organs, and urinary bladder were cut and stained with hematoxylin and eosin (H&E).

**Tests of response to a novel environment.**

The Open Field test was carried out at the GMC (11-week old mice) and at MGH (~10- and 40-week old mice) according to the standardized phenotyping screens developed by the Eumorphia partners [[3](#_ENREF_3)], available as EMPReSS protocols (see [www.eumodic.org](http://www.eumodic.org)). The Open Field apparatus/software used at GMC was ActiMot System from TSE systems, Bad Homburg, Germany. Open field starter package for mouse with Activity Monitor software used at MGH was from Med Associates, St. Albans, VT. The apparatus consisted of a transparent and infrared light permeable acrylic test arena with a smooth floor (internal measurements: 45.5 x 45.5. x 39.5 cm at GMC and 27.3 x 27.3 x 20.3 cm at MGH) and three-dimensional infrared light frame. For data analysis, the arena was divided by the software into two zones: the periphery defined as a corridor of 8 cm width along the walls and the center representing the remaining area of the arena. The center comprised 42% (GMC) or 17.1% (MGH) of the total arena. Illumination levels were set at approx. 150 lux in the corners and 200 lux in the middle of test arena. Animals were transported to the test room and left undisturbed for at least 30 minutes before the testing started. Then, each animal was placed individually into the middle of the right side of the arena facing the wall and allowed to explore freely for 20 min. In addition to infrared-beam detection, mouse movements were videotaped with tripod-mounted flip video camera for the first 10 minutes of each trial (MGH). After each trial, the test arena was cleaned with disinfectant. The following parameters were recorded for the whole arena, the periphery, and the center at 5-min intervals over the course of each 20-min trial: distance traveled (cm), resting, ambulatory, and vertical time (sec), rearing frequency, ambulatory counts (number of times mouse starts moving), stereotypy counts (number of times mouse exhibits stereotypic movement *e.g.* scratching, sniffing *etc.*) and stereotypy time (sec). Center zone entry frequency and latency to first enter the center zone (sec) were also measured by reviewing videotapes. Average velocity, percentage distance traveled, and percentage time spent were calculated for each zone at each time point from measured parameters.

**Light/dark box**

The test box was made of PVC and divided into two compartments, connected by a small tunnel (4 x 6 x 9 cm high). The lit compartment (29 x 19 x 24 cm high) was made of white PVC and was illuminated by cold light with an intensity of 650 lux in the middle; the dark compartment (14 x 19 x 24 cm high) was made of black PVC and not directly illuminated (approx. 20 lux in the centre). The mouse was placed in the centre of the black compartment and allowed to freely explore the apparatus for 5 min. Behaviors were observed by a trained observer sitting next to the box using a hand-held computer. Data were analyzed with respect to (1) the number of entries, latency to first entry, and time spent in both compartments and the tunnel; (2) the number of rearings in both compartments and the tunnel. Additionally, grooming behavior was recorded. An entry into a compartment was defined as placement of all four paws into the compartment. Additionally, a camera was mounted above the center of the test arena to videotape the trial, and the animal’s locomotor path in the lit compartment was analyzed with a video-tracking system (Ethovision 2.3, Noldus, Wageningen). The box was cleaned with disinfectant before each trial.

**Rotarod**

Rotarod analysis was performed according to standardized protocols [www.eumodic.org; [[3](#_ENREF_3)]]. Three trials with 15 minutes inter-trial-intervals were performed on an accelerating rotarod (4-40 rpm/5min; Bioseb, France at the GMC or Ugo Basile at MGH). For statistical analysis, a linear mixed effects model including sex, genotype, trial number and body mass was used (Software: S-Plus, Insightful).

**Social Discrimination**

The social discrimination test was carried out in the light phase according to [[4](#_ENREF_4)]. The experimental mouse was allowed to habituate to a testing cage for two hours. An ovariectomized (OVX) female mouse was then introduced to the testing cage with the experimental mouse. Behavior (contact with the OVX mouse) was recorded for 4 minutes, after which time the OVX female was removed. After two hours, the experimental mouse was again returned to the testing cage with the familiar OVX mouse as well as a novel unfamiliar OVX female. Behavior (contact with the novel and familiar conspecifics) was scored for another 4 minutes. Time spent in contact with the familiar and unfamiliar mice was compared using an unpaired Student’s t-test.

**Olfactory discrimination**

The olfactory discrimination test was carried out as published [[5](#_ENREF_5)]. Mice were placed on a restricted feeding regime to maintain their body weight at around 90% of their free-feeding level for the duration of training and testing. Experimental sessions were conducted 5 days per week, one session per day consisting of 12 -18 (GMC) or 12 (MGH) trials per day. The test was conducted using standard mouse cages (18.5 x 29.5 x 13 cm) with a small amount of bedding placed at the far end of the cage (GMC) or no bedding at all (MGH). The odorants used were ethyl trans-cinnamate (strawberry-like scent) or phenethylacetate (apple-like scent), both from Sigma. Each mouse was assigned to one odorant (“apple” or “strawberry”), and the odorants were randomized across the subjects in order to control for potential odor bias. The odorants were diluted to a concentration of 10% (v/v) in solvent (diethyl phthalate, Sigma) and presented either separately (for conditioning) or as binary mixtures (for testing) on fresh bedding shavings (ratio: 1ml of 10% solution per 3g shavings) in two circular, plastic dishes (3 cm diameter). The dishes were mounted horizontally on a carrier, separated by a vertical barrier between them. For each trial the carrier was inserted in the front part of the test cage. Between the trials, the mouse was separated from the front part by a barrier inserted by the experimenter. To begin a trial, the barrier was pulled out of the cage vertically, allowing for the mouse to move towards the dishes. To terminate a trial, the carrier with the dishes was removed from test cage. After each trial, the cage was cleaned with disinfectant. Every day, the panel of trials was performed at the same time (9 am to 2 pm). Conditioning procedure was performed in several steps: pre-training, training 1, and training 2. During pre-training, a single dish filled with clean, unscented bedding shavings was presented on each trial in the left or right location equally often (two identical dishes used at MGH). Mice were trained for three days to dig in any dish in order to retrieve a reward buried in the shavings (small piece of chocolate). In training step 1 the mice were trained to associate their assigned odor (designated [S+]) with the reward. One of the two dishes contained [S+]-scented shavings and the chocolate reward, while the other dish contained shavings with the solvent (diethylphthalate) and no reward. Correct choice was defined as first-choice digging in the dish with [S+]-scented shavings (mouse received the reward). Incorrect choice (digging in solvent dish) was followed by immediate termination of the trial (no reward). In training step 2, experimental conditions were the same as in training step 1, except the reward was buried in both [S+] and solvent dishes. Any training step was considered completed when each mouse made the correct choice in not less than 90% of all trials of the day. Following training, the mice were tested on their ability to discriminate between the two odorants ([S+] and [S-]) placed in two dishes with reward buried in both. Correct choice was defined as first-choice digging in the dish with [S+]-scented shavings. Then, the mice were tested on their ability to discriminate between the two odorants presented as pairs of [S+]:[S-] mixed in reverse proportions (70%:30% *vs.* 30%:70%; 55%:45% *vs.* 45%:55%; 53%:47% *vs.* 47%:53%; 51%:49% *vs.* 49%:51%; 50%:50% *vs.* 50%:50%. The previous testing step was taken as 100%:0 *vs.* 0%:100%.). Correct choice was defined as first-choice digging in the dish with the higher amount of [S+]. This was followed with reward given by experimenter with forceps. In case of incorrect choice, both dishes were immediately removed from the cage, terminating the trial. One binary dilution was tested per day in 18 trials (GMC) or 12 trials (MGH).

Testing of olfactory memory was carried out in the GMC pipeline of mice at 16-17 weeks and at 22 weeks after the initial olfactory discrimination test. The mice tested at 16-17 weeks were those that had been trained to recognize the strawberry odorant and those tested at 22 weeks were those that had been trained to recognize the apple odorant. The percentage of mice that recognized the [S+] odorant in a single trial was recorded.

**Gait analysis**

For automated gait analysis mice ran across a transparent runway in the dark, and their runs were video-tracked from below using an infrared-sensitive camera and the CatWalk software (Noldus, Wageningen, Netherlands). Per mouse, data of 3 runs with at least 5 consecutive step cycles were analyzed.

**Vertical pole descent**

Mice were tested for their ability to descend a vertical, metal threaded pole 50 cm in length (1 cm diameter), inserted into a platform. Each mouse was placed at the top of the bar with its head upwards. Animals were habituated to the apparatus for 3 days prior to testing. The time until the mouse turned around to climb down was recorded (t-turn), as well as the total time the mice needed to reach the floor with its forepaws (t-total). The test was repeated five times and for each session of five descents, the mean performance was recorded. If the mouse was not able to turn around completely, t-turn was defined as 60 seconds and t-total as 120 seconds. The same was done if an animal fell or slipped down the pole.

**Assessment of heart function**

Blood pressure was measured in unanesthetized mice with a non-invasive tail-cuff method using the MC4000 Blood Pressure Analysis Systems (Hatteras Instruments Inc., Cary, North Carolina, USA). Surface limb ECG was performed in a Faraday cage on anesthetized mice using metal bracelets positioned on the front-paws and the left hind-paw. ECG was recorded for about seven minutes and the traces analyzed using ECG-auto software (EMKA technologies, Paris, France). Plasma Nt-proANP concentrations were quantified using a commercial Nt-proANP enzyme-linked immunosorbent assay (Biomedica Medizinprodukte, Vienna, Austria). Heart weight was determined following exsanguination by cutting the dorsal aorta, removal of the heart from the pericardial membrane, cutting the major vessels at the point they enter or exit the atria and blotting the organ on paper towels. The tibia length was determined from the left tibia of the mouse using a ruler. Heart function was analyzed in an additional cohort of mice at 69 weeks of age. Echocardiogram was performed as previously described [[6](#_ENREF_6)]. Briefly, mouse was anesthetized with isoflurane via vaporizer and all measurements were obtained using a Vevo 770 High-Resolution In Vivo Micro-Imaging System and an RMV 707B Scanhead (VisualSonics Inc.). Three consecutive beats at heart rate between 500 and 550 beats per minute were averaged in each measurement. Ambulatory ECG recordings and in vivo electrophysiological studies were performed as described previously [[7](#_ENREF_7)].

**Assessment of lung function**

Breathing patterns were assessed using whole body plethysmography (Buxco® Electronics, Sharon, Connecticut) in unrestrained animals. Measurements were performed between 8 a.m. and 11 a.m. to account for potential diurnal variations in breathing in a quiet room where temperature and humidity were kept constant throughout the measurements. Before each measurement, the system was calibrated and the actual barometric pressure, temperature, and humidity were supplied to ensure adequate calculations of flow rates and volumes. After placing the animals into the chamber, data recording was immediately started and was continued for 40 min. Mice underwent typical phases during the measuring period. Following an ~5 minute period of stress and high respiratory rate the animals entered a phase of activity characterized by exploration and grooming and a slightly lower respiratory rate. This was followed by a resting phase in which activity was more and more interrupted by rest or short periods of snoozing or sleep, resulting in a further marked decrease in respiratory rate.

**Intraperitoneal glucose tolerance test**

Before the test food was removed for 16 to 18 hours overnight and the baseline fasting blood glucose level was determined from a small drop of blood obtained from the tip of the tail, scored using a sterilized scalpel blade, using the Accu-Chek Aviva glucose analyzer (Roche/ Mannheim). Thereafter mice were injected intraperitoneally with 2 g of glucose/kg body weight using a 20% glucose solution. 15, 30, 60, 90 and 120 minutes after glucose injection, a blood sample was obtained and analyzed as above.

**Analysis of clinical chemistry parameters**

For the determination of fasting blood lipid and glucose values, food was withdrawn for 16 to 18 hours over night before blood collection. Blood samples obtained by retro-orbital sinus puncture were collected from unfed and *ad libitum* fed mice in Li-heparin coated tubes. Plasma was separated in a centrifugation step (10 min, 5000 x g; 8°C) Biofuge fresco, Heraeus; Hanau, Germany) and plasma samples obtained from *ad libitum* fed mice were diluted 1: 2 with aquadest, mixed and then centrifuged again for 10 min at 5000 x g at 8°C. Clinical chemistry parameters were determined using an Olympus AU 400 autoanalyzer and adapted reagents from Olympus (Hamburg, Germany) except creatinine that was measured using an enzymatic kit from Biomed (Oberschleißheim, Germany) and free fatty acids (NEFA) that were measured using a kit from Wako Chemicals GmbH (Neuss, Germany).

**References**

1. Fuchs H, Schughart K, Wolf E, Balling R, Hrabe de Angelis M (2000) Screening for dysmorphological abnormalities--a powerful tool to isolate new mouse mutants. Mammalian genome : official journal of the International Mammalian Genome Society 11: 528-530.

2. Fuchs H, Lisse T, Abe K, Hrabe de Angelis M (2006) Screening for bone and cartilage phenotypes in mice. In: Hrabe de Angelis M, Chambon P, Brown S, editors. Phenotyping of the Laboratory Mouse. Weinheim: Wiley-VCH. pp. 35-86.

3. Mandillo S, Tucci V, Holter SM, Meziane H, Banchaabouchi MA, et al. (2008) Reliability, robustness, and reproducibility in mouse behavioral phenotyping: a cross-laboratory study. Physiol Genomics 34: 243-255.

4. Feil R, Holter SM, Weindl K, Wurst W, Langmesser S, et al. (2009) cGMP-dependent protein kinase I, the circadian clock, sleep and learning. Commun Integr Biol 2: 298-301.

5. Deussing JM, Breu J, Kuhne C, Kallnik M, Bunck M, et al. (2010) Urocortin 3 modulates social discrimination abilities via corticotropin-releasing hormone receptor type 2. J Neurosci 30: 9103-9116.

6. Chen PC, Wakimoto H, Conner D, Araki T, Yuan T, et al. (2010) Activation of multiple signaling pathways causes developmental defects in mice with a Noonan syndrome-associated Sos1 mutation. The Journal of clinical investigation 120: 4353-4365.

7. Wakimoto H, Kasahara H, Maguire CT, Izumo S, Berul CI (2002) Developmentally modulated cardiac conduction failure in transgenic mice with fetal or postnatal overexpression of DNA nonbinding mutant Nkx2.5. Journal of cardiovascular electrophysiology 13: 682-688.
